# New material of the ‘microsaur’ *Llistrofus* from the cave deposits of Richards Spur, Oklahoma and the paleoecology of the Hapsidopareiidae

**DOI:** 10.7717/peerj.6327

**Published:** 2019-01-25

**Authors:** Bryan M. Gee, Joseph J. Bevitt, Ulf Garbe, Robert R. Reisz

**Affiliations:** 1 Department of Biology, University of Toronto Mississauga, Mississauga, ON, Canada; 2 Australian Centre for Neutron Scattering, Australian Nuclear Science and Technology Organisation, Lucas Heights, NSW, Australia; 3 International Centre of Future Science, Jilin University, Changchun, Jilin Province, China

**Keywords:** *Llistrofus*, Microsaur, Permian, Hapsidopareiidae, Recumbirostra, Lepospondyli

## Abstract

The Hapsidopareiidae is a group of “microsaurs” characterized by a substantial reduction of several elements in the cheek region that results in a prominent, enlarged temporal emargination. The clade comprises two markedly similar taxa from the early Permian of Oklahoma, *Hapsidopareion lepton* and *Llistrofus pricei*, which have been suggested to be synonymous by past workers. *Llistrofus* was previously known solely from the holotype found near Richards Spur, which consists of a dorsoventrally compressed skull in which the internal structures are difficult to characterize. Here, we present data from two new specimens of *Llistrofus*. This includes data collected through the use of neutron tomography, which revealed important new details of the palate and the neurocranium. Important questions within “Microsauria” related to the evolutionary transformations that likely occurred as part of the acquisition of the highly modified recumbirostran morphology for a fossorial ecology justify detailed reexamination of less well-studied taxa, such as *Llistrofus*. Although this study eliminates all but one of the previous features that differentiated *Llistrofus* and *Hapsidopareion*, the new data and redescription identify new features that justify the maintained separation of the two hapsidopareiids. *Llistrofus* possesses some of the adaptations for a fossorial lifestyle that have been identified in recumbirostrans but with a lesser degree of modification (e.g., reduced neurocranial ossification and mandibular modification). Incorporating the new data for *Llistrofus* into an existing phylogenetic matrix maintains the Hapsidopareiidae’s (*Llistrofus* + *Hapsidopareion*) position as the sister group to Recumbirostra. Given its phylogenetic position, we contextualize *Llistrofus* within the broader “microsaur” framework. Specifically, we propose that *Llistrofus* may have been fossorial but was probably incapable of active burrowing in the fashion of recumbirostrans, which had more consolidated and reinforced skulls. *Llistrofus* may represent an earlier stage in the step-wise acquisition of the derived recumbirostran morphology and paleoecology, furthering our understanding of the evolutionary history of “microsaurs.”

## Introduction

The karst deposits near Richards Spur, Oklahoma preserve a diverse early Permian tetrapod assemblage that includes the recumbirostran “microsaurs” *Cardiocephalus peabodyi* (Carroll & Gaskill, 1978) and *Nannaroter mckinziei* (Anderson, Scott & Reisz, 2009). Isolated tooth-bearing elements previously associated with the recumbirostran *Euryodus primus* (Gregory, Peabody & Price, 1956) were recently recognized to belong to the captorhinid *Opisthodontosaurus* (Reisz et al., 2015). This was interpreted as evidence of a high degree of mandibular convergence with the “microsaur.” Similarly, *Bolterpeton carrolli* was recently demonstrated to be a junior synonym of the parareptile *Delorhynchus* (Haridy, MacDougall & Reisz, 2017). The assemblage also includes the “microsaur” *Llistrofus pricei* (Carroll & Gaskill, 1978; Bolt & Rieppel, 2009). That taxon is recognizable by a large, ventrally open temporal emargination, which results from a reduction in the jugal, the postorbital, and the squamosal. This emargination is shared with another early Permian “microsaur,” *Hapsidopareion*, and unites them within the Hapsidopareiidae (Bolt & Rieppel, 2009). *Llistrofus* is differentiated from *Hapsidopareion* (from the nearby early Permian South Grandfield locality) on the basis of a much larger skull, a frontal that contacts the orbit, and a cultriform process that is off-set from the main body of the parasphenoid (Bolt & Rieppel, 2009). Both taxa are endemic to early Permian deposits of Oklahoma, with *Llistrofus* known from Richards Spur and *Hapsidopareion* known from South Grandfield (Daly, 1973; Bolt & Rieppel, 2009). This clade has sometimes included the early Permian taxon *Saxonerpeton* from Germany, despite the absence of an emargination in that taxon (Carroll & Gaskill, 1978). However, more recent phylogenetic analyses recovered *Saxonerpeton* as sister to the Hapsidopareiidae, which in turn is recovered as the earlier diverging sister group to the recumbirostran “microsaurs” (Ruta, Jeffery & Coates, 2003; Huttenlocker et al., 2013; Pardo et al., 2017). Recumbirostrans are characterized by a diverse array of shared adaptations for a fossorial ecology (e.g., increased neurocranial ossification, mandibular modifications). Many taxa have been described through computed tomographic (CT) analyses that permit the study of internal structures (particularly the neurocranium) (Maddin, Olori & Anderson, 2011; Huttenlocker et al., 2013; Pardo, Szostakiwskyj & Anderson, 2015; Szostakiwskyj, Pardo & Anderson, 2015; Pardo & Anderson, 2016) that have historically received less attention due to the inaccessible nature of these regions. However, these forms represent markedly specialized morphotypes suggested to have been adapted for a variety of burrowing behaviors. The earlier stages of “microsaur” evolution, both in general and with respect to the acquisition of the recumbirostran suite of characters, remain poorly understood.

**Figure 1:**
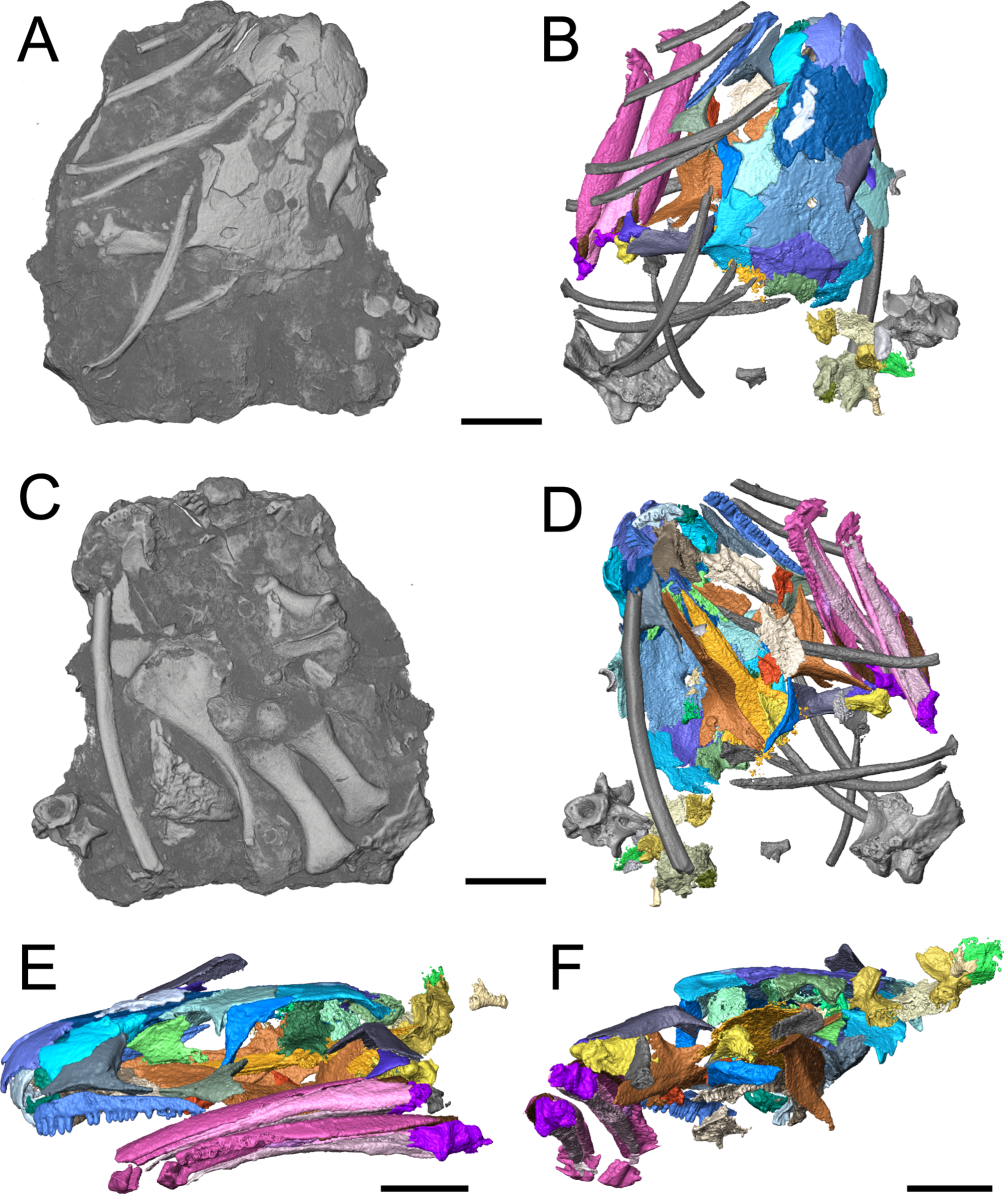
Referred specimen of *Llistrofus pricei* (OMNH 79031). (A) Volumetric rendering of the specimen in dorsal profile; (B) segmented visualization in the same profile of all cranial elements interpreted as belonging to *L. pricei*; (C) volumetric rendering in ventral profile; (D) segmented visualization in the same profile; (E) segmented visualization without postcrania in left lateral profile; (F) segmented visualization without postcrania in posterior profile. The color palette follows the division of the description: blues - skull roof oranges/browns—palate; reds/purples—mandibles; greens—occiput/otic capsule. Labeled figures with a focus on different skeletal regions are presented in Figs. 2–10. Scale bars equal to four mm.

The holotype (FM UR 948) of *Llistrofus pricei*, comprising a skull with mandibles in articulation with a partial vertebral column and other disarticulated postcrania, was described by Carroll & Gaskill (1978); the cranial material was redescribed by Bolt & Rieppel (2009) following additional mechanical preparation. The dorsoventrally compressed holotype was the only described specimen of *Llistrofus*, and as a result, important aspects of the cranial morphology remained unresolved. Furthermore, the functional significance of the unusually large temporal emargination and its implications for the paleoecology of the taxon were not addressed in great detail by past workers. The new material that we present here consists of two partial skulls, each associated with partial to complete mandibles, and postcrania associated with one of the skulls. One of these specimens consists of a dense assortment of elements in a block and was analyzed using neutron tomography (NT) that revealed additional anatomical details (Fig. 1).

The integration of NT into paleontology has been limited to date (see Cisneros et al., 2010; Grellet-Tinner et al., 2011; Laaß et al., 2011; Martins et al., 2011; Salvemini et al., 2016; De Beer, 2017; Louys et al., 2017 for examples of previous studies), and the analysis featured in this study provides important data regarding its utility, particularly for material from Richards Spur. These specimens contribute new data regarding the morphology and sutural patterns of some of the less well-preserved regions of the skull roof (e.g., premaxilla), the palate (e.g., parasphenoid, ectopterygoid), the neurocranium (e.g., pleurosphenoid, orbitosphenoid), and the otic capsule (stapes, opisthotic, prootic). These data permit a more thorough exploration of the potential functional and evolutionary drivers of the temporal emargination (e.g., heterochrony, miniaturization, fossorial ecology). *Llistrofus* shares some characteristics with recumbirostrans that are associated with fossoriality (e.g., orbitosphenoids that contact the skull roof, well-developed retroarticular process) but with a lower degree of overall ossification than in recumbirostrans (e.g., absence of anterior median ossifications of the braincase). *Llistrofus* may thus represent an earlier, more generalized intermediate in the evolutionary trajectory toward the specialized recumbirostran morphology.

## Materials and Methods

### Materials

Two newly referred skulls (OMNH 73718, OMNH 79031) and one referred isolated rib (OMNH 79032) are described in this study. OMNH 73718 consists of a partial skull roof, preserved anteriorly, with a complete, articulated left mandible and a partial, articulated right mandible. Only a small portion of the right palate and the elements of the right postorbital region (postorbital, postfrontal, jugal, squamosal) are preserved. OMNH 79031 consists of a complete skull with an associated but partially disarticulated palate, complete but disarticulated occiput, partial otic capsules, partial neurocranium, pair of complete but disarticulated mandibles, partial atlas-axis complex, and tentatively associated trunk vertebrae and ribs. The block also contains a large number of fragments both within and around the skull roof of *Llistrofus*, much of which is skeletally and taxonomically indeterminate. Many of these elements, both those belonging to *Llistrofus* and those that cannot be identified, were not visible externally and could only be studied through CT. Most elements of *Llistrofus* remain in relative association or articulation. OMNH 79032 is an isolated rib that is tentatively referred to *Llistrofus* on the basis of striations on the external surface that are unknown among other “microsaurs” or other tetrapods from Richards Spur and is included in order to illustrate these striations. No permits were required for the described study, which complied with all relevant regulations.

### Methods

OMNH 73718 and OMNH 79031 were prepared using a pin vise and air scribes. Both were photographed prior to any further analyses. OMNH 79031 was additionally imaged using the NT methods outlined below. The isolated rib (OMNH 79032) was imaged using a Neoscope JCM-5000 scanning electron microscope (Jeol, Peabody, MA, USA). Figures were produced using Adobe Illustrator and Photoshop CS6.

### Neutron imaging

This study utilized the DINGO radiography/tomography/imaging station, located on the thermal HB 2 beam, tangentially facing the 20 MW Open-Pool Australian Lightwater (OPAL) reactor housed at the Australian Nuclear Science and Technology Organisation, Lucas Heights, New South Wales, Australia. The DINGO facility utilizes a quasi-parallel collimated beam of thermal neutrons from OPAL with a maximum spectrum intensity at 1.08 Å (70 meV), full-width-at-half-maximum of 0.9 Å (100 meV), and two collimation (*L*/*D*) ratios of 500 or 1,000 (Garbe et al., 2015), where *L* is the neutron aperture-to-sample length and *D* is the neutron aperture diameter. For the measurement described here, an *L*/*D* ratio of 1,000 was used to ensure highest available spatial resolution; all details of scanning specifications are included with the raw data (http://morphobank.org/permalink/?P3134).

Neutrons were converted to photons using a 100 × 100 0.05 mm ZnS(Ag)/^6^LiF scintillator screen and resultant photons detected by an Andor IKON-L CCD camera (liquid cooled, 16-bit, 2,048 × 2,048 pixels) coupled with a Makro Planar 100 mm Carl Zeiss lens and 30 mm extension tube. A total of 1,001 equally-spaced angle shadow-radiograph projections were obtained every 0.18° as the sample was rotated over 180° about its vertical axis. Both dark (closed shutter) and beam profile (open shutter) images were obtained for calibration before initiating shadow-radiograph acquisition. A cosmic ray filter was applied to all images to reduce data noise associated with non-neutron background radiation detection events. To further reduce anomalous noise, a total of three individual radiographs with an exposure length of 60 s were acquired at each angle. These individual radiographs were summed in postacquisition processing using the “Grouped ZProjector” plugin in ImageJ v.1.51h (National Institutes of Health, Bethesda, MD, USA); this plugin was developed by Holly (2004). Tomographic reconstruction of the 16-bit raw data was performed using Octopus Reconstruction v.8.8 (Inside Matters NV), yielding a voxel size of 16.1 × 16.1 × 16.1 μm and virtual slices perpendicular to the rotation axis. When these slices are stacked in a sequence, they form a three-dimensional volume image of the sample. Unprocessed 16-bit TIFF slices are available online through MorphoBank (project #3134; http://morphobank.org/permalink/?P3134) (O’Leary & Kaufman, 2012) and upon request from the Sam Noble Oklahoma Museum of Natural History (OMNH). The reconstructed volume data were downsampled by a factor of 2 in ImageJ to reduce computation time and then rendered and segmented using Avizo Lite 9.3.0. The supplemental animation was generated by importing frames of the segmented data into ImageJ.

### Phylogenetic analysis

In order to contextualize the more fully resolved anatomy of *Llistrofus*, we coded the taxon into two different data matrices. The data matrix of Huttenlocker et al. (2013) has broad taxonomic sampling of “microsaurs,” including *Hapsidopareion* and *Saxonerpeton*, but some of the characters and codings are outdated in light of more recent work (especially tomographic studies) of recumbirostrans. This matrix was primarily utilized to assess the interrelationships of *Llistrofus, Hapsidopareion*, and *Saxonerpeton*. We also used the data matrix of Pardo et al. (2017), which has increased character sampling and updated coding but that does not include *Hapsidopareion* and *Saxonerpeton* in order to test whether the new data changed the relationship of *Llistrofus* to other “microsaurs” (predominantly to recumbirostrans). Neither *Hapsidopareion* nor *Saxonerpeton* are coded in the Pardo et al. (2017) matrix, and without having personally observed specimens of these taxa, we did not attempt to code them ourselves. Both matrices were analyzed using the original parameters of the previous studies (Huttenlocker et al., 2013:549; Pardo et al., 2017:646) using PAUP* version 4.0a (build 164) for Macintosh. Revised character coding for *Llistrofus* is included as a Supplemental File.

## Systematic Paleontology

Tetrapoda Goodrich, 1930
Lepospondyli Zittel, 1888
Microsauria Dawson, 1863
Hapsidopareiidae *sensu* Bolt & Rieppel, 2009

**Revised diagnosis.** “Microsaurs” with a large temporal emargination that is open ventrally and separated from the orbit. The jugal and postorbital form a narrow postorbital bar while the squamosal is reduced to a narrow vertical bar, with nearly parallel anterior and posterior margins. The emargination extends dorsally to the level of the lateral margin of the tabular. The quadratojugal, if present, does not contact the jugal, and there is no jugal–squamosal contact.

**Discussion.** As noted by Marjanović & Laurin (2019), the family-level nomenclatural derivation of Hapsidopareiontidae by Daly (1973) does not conform to the regulations designated by the International Commission on Zoological Nomenclature (ICZN) and should be amended to Hapsidopareiidae (see articles 29.1–29.3 for the relevant guidelines). This correction does not require a formal ICZN opinion and thus is followed here.

*Llistrofus sensu* Bolt & Rieppel, 2009
*Llistrofus pricei* Carroll & Gaskill, 1978

**Holotype.** FM UR 948, partial skull, vertebral column, ribs, ulna, radius, and scales.

**Horizon and locality.** Karst infills in the Dolese Brothers Limestone Quarry (Sakmarian, Permian), SW1/4, sec. 31, T4N, R11W, Comanche Co., Oklahoma.

**Referred material.** OMNH 73718, partial skull with articulated partial mandibles; OMNH 79031, partial skull with disarticulated mandibles, palate, and braincase, vertebrae, ribs, and scales; OMNH 79032, isolated rib.

**Revised diagnosis.** Hapsidopareiid “microsaur” characterized by a frontal that enters the orbital margin, a prefrontal that contacts the posterior narial margin, a premaxilla that contributes to the ventral narial margin, exclusion of the postfrontal from the temporal emargination, a tabular that contacts the postorbital, the presence of denticles on the vomer, the presence of teeth on the palatine along the posteroventral margin of the choana that are smaller than the marginal teeth, absence of a pterygoid–premaxilla contact, a splenial that contributes to the mandibular symphysis, the presence of a Meckelian foramen, and the presence of a retroarticular process.

**Discussion.** The skull size of *Llistrofus*, which is twice that of any known specimen of *Hapsidopareion*, was previously included in the diagnosis. However, relative size should not be considered a reliable feature for species discrimination given the high degree of morphological similarity between these taxa and because an ontogenetic influence cannot be ruled out. A separation of the cultriform process from the basal plate of the parasphenoid was previously included as a diagnostic feature but is here demonstrated to be the result of taphonomic damage. As noted by previous workers (Bolt & Rieppel, 2009), the presence of a small quadratojugal in *Llistrofus* may be another differential feature if the absence of this element in *Hapsidopareion* is not a taphonomic artifact. It should be noted that Bolt & Rieppel (2009) identified 17 characters that would differentiate the two taxa, based on the description of each by Carroll & Gaskill (1978) but considered almost all of them to be highly suspect or somewhat arbitrary due to the poor condition of material of *Hapsidopareion*.

**Figure 2:**
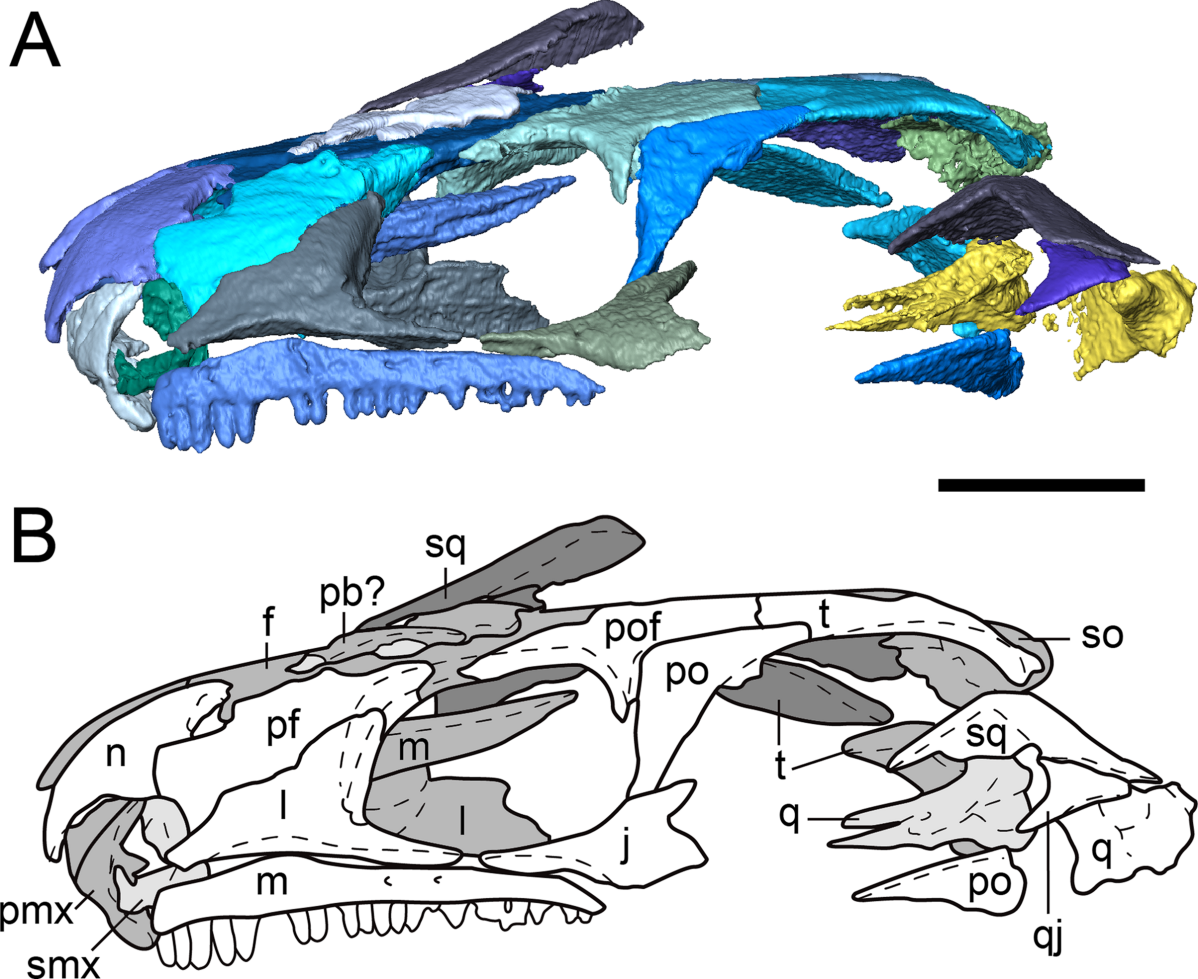
Partial dorsal skull roof of referred specimen of *Llistrofus pricei* (OMNH 79031) in left lateral profile. (A) Segmented visualization of the skull roof; (B) outline drawing of the skull roof. Refer to Fig. 1 caption for color palette. Abbreviations: f, frontal; j, jugal; l, lacrimal; m, maxilla; n, nasal; p, parietal; pb, palpebral bone; pf, prefrontal; pmx, premaxilla; po, postorbital; pof, postfrontal; pp, postparietal; q, quadrate; qj, quadratojugal; smx, septomaxilla; so, supraoccipital; sq, squamosal; t, tabular. Scale bars equal to four mm.

**Figure 3:**
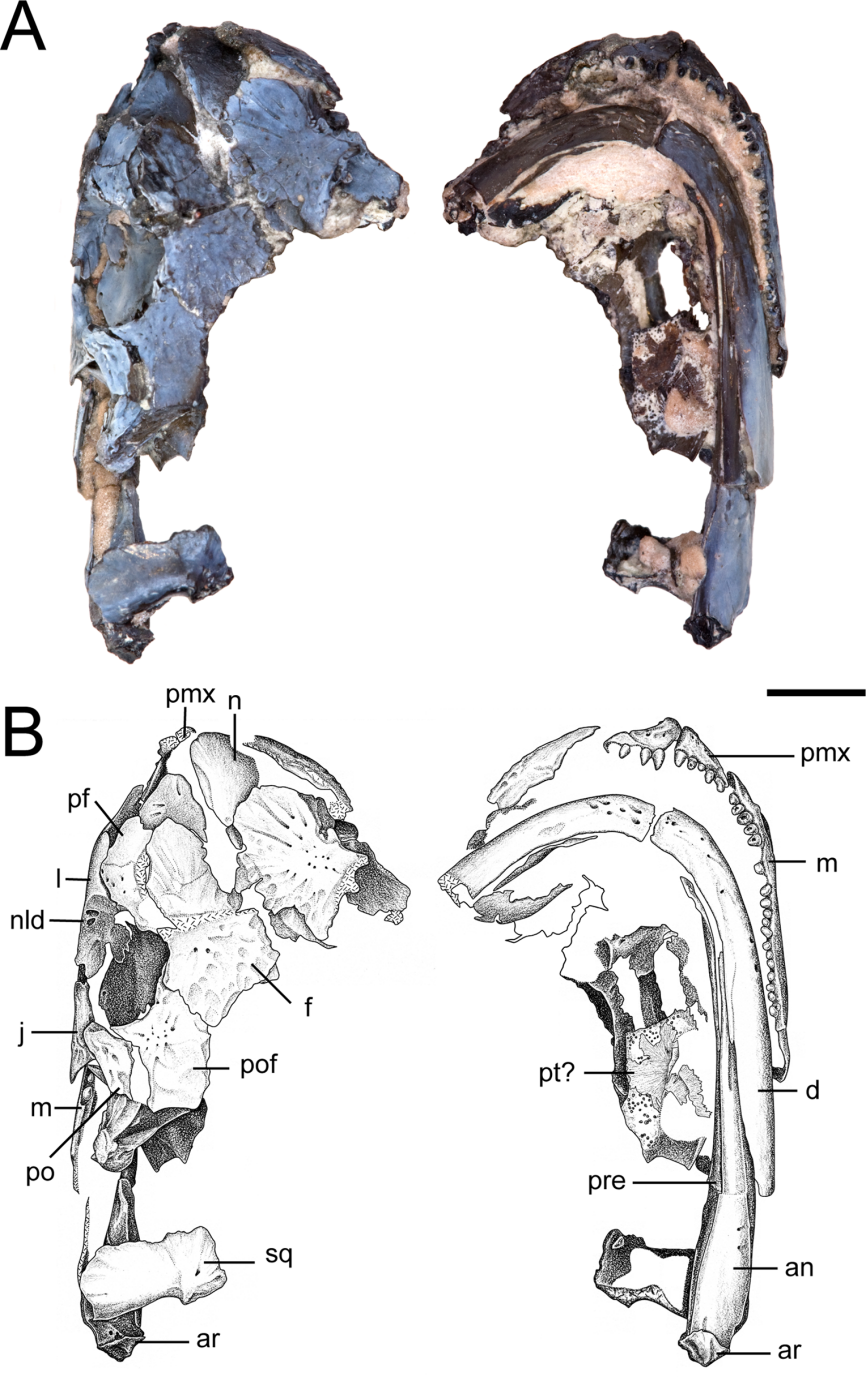
Partial skull of referred specimen of *Llistrofus pricei* (OMNH 73718). (A) Photographs of the skull in dorsal and ventral profiles; (B) illustrations of the skull in dorsal and ventral profiles. Abbreviations: an, angular; ar, articular; d, dentary; f, frontal; j, jugal; l, lacrimal; m, maxilla; n, nasal; nld, external expression of the nasolacrimal duct; pf, prefrontal; pmx, premaxilla; po, postorbital; pof, postfrontal; pre, prearticular; pt, pterygoid; qj, quadratojugal; sq, squamosal. Scale bar equal to four mm. Photo credit: Diane Scott; illustration credit: Nicola Horsman.

**Figure 4:**
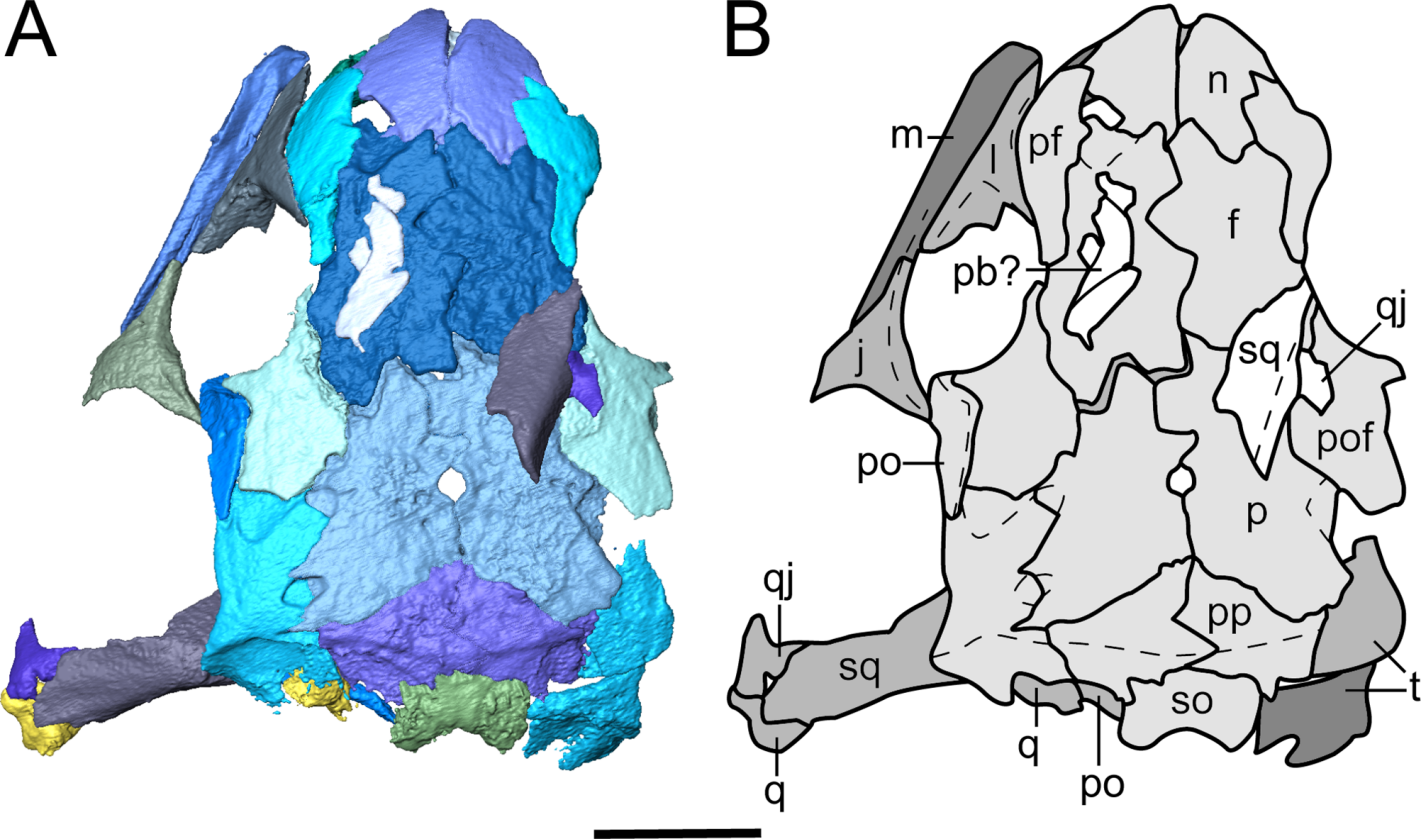
Segmented skull roof of referred specimen of *Llistrofus pricei* (OMNH 79031) in dorsal profile. (A) Segmented visualization of the skull roof; (B) outline drawing of the skull roof. Abbreviations and color palette as with Fig. 2. Scale bar equal to four mm.

**Figure 5:**
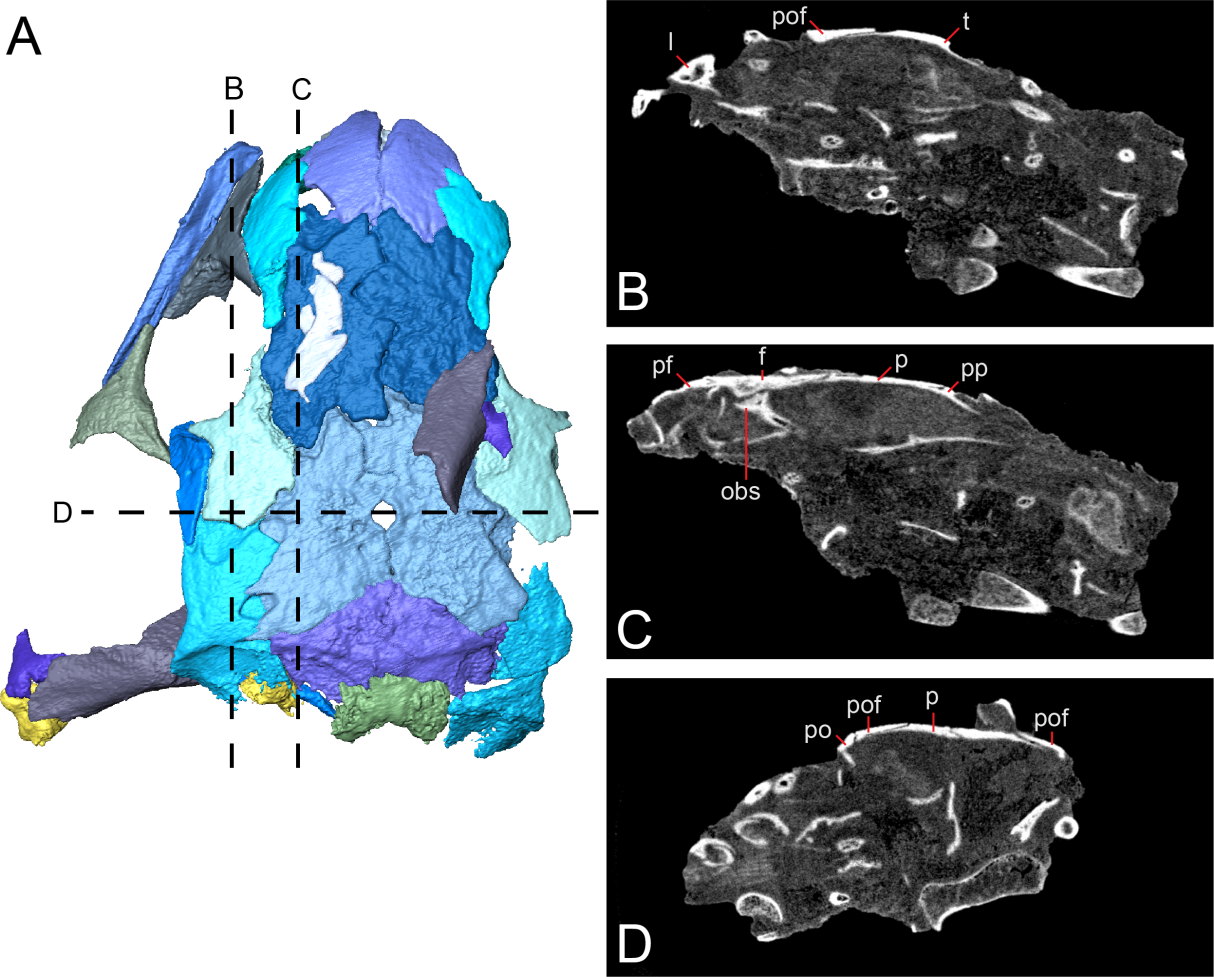
Tomographic slices through the referred specimen of *Llistrofus pricei* (OMNH 79031) showing sutural relationships of the cranial elements. (A) Segmented visualization of the skull roof (from Fig. 4); (B and C) longitudinal slices; (D) transverse slice. Abbreviations: f, frontal; l, lacrimal; obs, orbitosphenoid; p, parietal; po, postorbital; pof, postfrontal; pp, postparietal; t, tabular.

**Description.** The skull of *Llistrofus* is moderately tall and box-like in lateral view, with the dorsal surface curving ventrally at the snout (Fig. 2). In dorsal view, the skull is subtriangular and tapers gradually anterior to the temporal emargination into a rounded snout (Figs. 3 and 4). Many of the cranial sutures are joints formed by extensive underplating of adjacent elements by thin flanges, features that are seen in other “microsaurs” (e.g., Szostakiwskyj, Pardo & Anderson, 2015; Fig. 5). In OMNH 73718, the anterior portions of the skull, articulated with the mandibles, are preserved but dorsoventrally compressed (Fig. 3). The exposed surfaces of OMNH 79031 are limited to the dorsal surface and part of the left temporal region of the skull (Fig. 1A; Movie S1). Examination of the specimen using NT revealed additional profiles of exposed elements and a large number of obscured elements, including paired mandibles, the premaxillae and maxillae, the entirety of the palate, the neurocranium, occiput, and otic capsules, and some postcrania (Figs. 1B and 1D–1F; Movie S1). Some disarticulation has occurred in the right side of the skull, with many of the temporal and palatal elements being displaced to the left side, and in the occiput, shifted posteriorly and to the right. Minor dislodgement of the rostrum (premaxillae, right septomaxilla), the anterior braincase (orbitosphenoids), and a left shift of the mandibles is also noted. The overall size of both skulls is comparable to that of the holotype (Table 1). The following description is considered representative of both specimens, with deviations noted where appropriate, and focuses primarily on a comparison with the closely related *Saxonerpeton* (Carroll & Gaskill, 1978) and *Hapsidopareion* (Daly, 1973; Carroll & Gaskill, 1978). Features of the neurocranium are compared to those in recumbirostrans, primarily because a comparable CT dataset is not available for non-recumbirostran “microsaurs.”

**Table 1:** Measurements for the two new referred skulls of Llistrofus in relation to the holotype.

Measurement	FM UR 948	OMNH 79031	OMNH 73718
Total skull length	>1.81	2.02	>2.09*
Frontal to postparietal	1.63	1.59	–
Frontal-anterior orbital margin	–	0.25	0.16
Frontal-posterior orbital margin	–	0.82	0.69
Interorbital	0.63	0.75	0.81
Frontal-anterior margin of pineal foramen	0.88	0.99	–

**Notes:**

All measurements of the newly referred specimens were made based on digital photographs and are given in centimeters. Measurements of the holotype were made using the figures of Bolt & Rieppel (2009).

* Approximated via mandible length.

**Figure 6:**
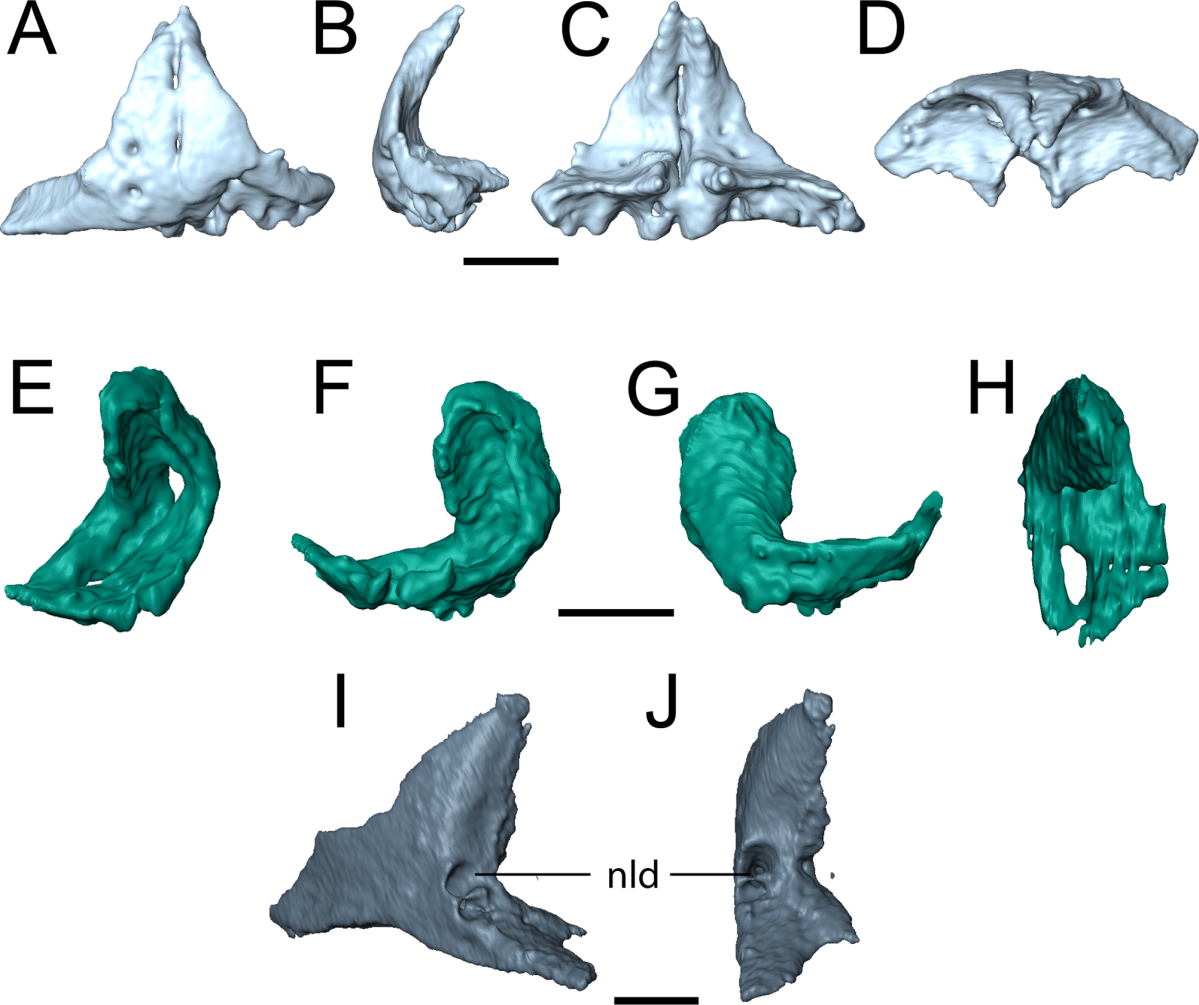
Selected profiles of the selected snout elements of referred specimen of *Llistrofus pricei* (OMNH 79031). (A) isolated premaxillae in anterior profile; (B) the same in left lateral profile; (C) the same in posterior profile; (D) the same in dorsal profile; (E) isolated left septomaxilla in anterolateral profile; (F) the same in lateral profile; (G) the same in medial profile; (H) the same in dorsal profile; (I) isolated left lacrimal showing external expression of the nasolacrimal duct in posterolateral profile; (J) the same in posterior profile. Abbreviation: nld, external expression of the nasolacrimal duct. Scale bars equal to one mm.

**Snout.** The premaxilla is a subtriangular element that is preserved in both new specimens but not in the holotype (Figs. 3, 4 and 6A–6D). Presumably, the dorsal process would have contacted the nasal to contribute to the margin of the external naris, but this contact is not articulated in either specimen. The dorsal process is slightly convex in OMNH 79031 (Fig. 6B) and tapers to a point that could represent an alary process (Fig. 6D), as in *Hapsidopareion* (Daly, 1973). However, without articulation, it is not possible to confidently discern the degree of dorsal exposure or whether the snout would have been recumbent. The position of the teeth, relatively posteroventral to the main dorsoventral axis of the premaxilla, suggests that the inflection point of the snout would have been within this element, not at the junction with the nasal. Internally, each premaxilla includes a triangular posteriorly directed process near the ventral margin; these processes frame a small, oval opening (Fig. 6D). Based on OMNH 73718, the premaxilla is sutured to the maxilla via a short, overlapping posterior process (Fig. 3B). As suggested by Bolt & Rieppel (2009), both elements contribute to the ventral margin of the naris, in contrast to the exclusive contribution of the maxilla reconstructed by Carroll & Gaskill (1978:fig. 16). Five premaxillary tooth positions are found in both specimens, a count shared with many other “microsaurs,” including *Hapsidopareion* (Daly, 1973) and *Saxonerpeton* (Carroll & Gaskill, 1978) and the recumbirostrans *Micraroter* (Carroll & Gaskill, 1978), *Tambaroter* (Henrici et al., 2011), (Henrici et al., 2011), and *Proxilodon*, and *Huskerpeton* (Huttenlocker et al., 2013). A few small foramina are found on the anterior surface of the premaxilla in both specimens (Figs. 3, 6A and 6C); in OMNH 79031, these can be seen to exit through the posterior surface at about the same dorsoventral and mediolateral position (Fig. 6C). Each premaxilla seems to carry two foramina, although only one is resolved in the right premaxilla of OMNH 79031 through the tomographic data.

The maxilla is sutured to the premaxilla anteriorly, to the lacrimal dorsally, and to the jugal posteroventrally (Figs. 2–4). There is a broad dorsal groove anteriorly to accommodate the lacrimal that narrows posteriorly for the jugal and that terminates in a small foramen. The contribution of the maxilla to the orbital margin is not confidently identifiable in any specimen of *Llistrofus*, but as suggested by past authors (Carroll & Gaskill, 1978; Bolt & Rieppel, 2009; though contrary to the generic diagnosis of the latter), a lacrimal–jugal contact likely excluded the maxilla; there is no evidence for a dorsal expansion of the maxilla that could be identified as part of the margin. Teeth occur along the entire length of the maxilla. A maximum tooth count of 21 was identified in each specimen, in agreement with the count made by Bolt & Rieppel (2009), which was revised from the 20 positions noted by Carroll & Gaskill (1978). In contrast, most “microsaurs” possess fewer than twenty maxillary teeth (Carroll & Gaskill, 1978:table 3). This is three more than in *Hapsidopareion* and one fewer than in *Saxonerpeton* (Carroll & Gaskill, 1978; Bolt & Rieppel, 2009). The maxillary tooth count in *Hapsidopareion* is less certain; although Carroll & Gaskill (1978:table 3) list 18 positions, the description by Daly (1973) suggested that 20 positions was not unreasonable.

The teeth are small with rounded tips and pleurodont implantation, formed by a taller labial wall. There is no apparent pattern of replacement (e.g., alternating positions) in either specimen; both possessed the vast majority of the marginal dentition at the time of death. The premaxillary teeth decrease gradually in size posteriorly. The maxillary teeth in OMNH 79031 initially increase in size, with the fifth position on the right element being the largest; its correlative position on the left element is absent. Posterior to this position, the teeth gradually decrease in size. It is noteworthy that on the right maxilla, the fifth position is followed by a vacant socket, whereas in the left maxilla, the fifth position is vacant but followed by the largest tooth of the row in the sixth position. This may be a naturally enlarged tooth (“caniniform”), rather than a tooth that appears larger due to the presence of recently replaced adjacent teeth, but no enlarged tooth has been noted in the holotype, and this tooth is not as disproportionately enlarged as in the gymnarthrid *Euryodus primus*, for example (Olson, 1939). In OMNH 73718, no maxillary tooth position appears enlarged. The enlarged tooth of OMNH 79031 may thus be the product of intraspecific variation. There is no documentation of an enlarged tooth in *Hapsidopareion* or *Saxonerpeton* (Daly, 1973; Carroll & Gaskill, 1978). Curiously, Bolt & Rieppel (2009:481, fig. 4) reconstructed the marginal dentition of *Llistrofus* as increasing to the third position, decreasing and remaining constant for six tooth positions, increasing again and remaining more or less constant for another six tooth positions, and finally decreasing at the posterior terminus of the element. This more irregular pattern differs greatly from that noted in our observations but was not discussed, nor can it be confirmed from photographs and line drawings of the holotype (Bolt & Rieppel, 2009:fig. 2).

The holotype of *Llistrofus* lacks a septomaxilla, which was cited as a feature shared with *Saxonerpeton* and in contrast to *Hapsidopareion* (Daly, 1973; Carroll & Gaskill, 1978; Bolt & Rieppel, 2009). However, a well-ossified septomaxilla is present in OMNH 79031 in the left naris in the form of a slender, curved element at the posterior margin (Figs. 2 and 6E–6H), which resembles that of *Hapsidopareion*. The right septomaxilla is dislodged and adhered to the ventral surface of the right prefrontal and nasal. The element forms an inverted C-shape in lateral profile (Figs. 6F and 6G). The internal surface is concave, with the ventral surface formed by two parallel, anteroposteriorly directed termini. The termini extend posteriorly, where they meet to enclose the opening for the lacrimal duct. A single process then ascends dorsally and curves anteriorly along the dorsal narial margin (Figs. 6E and 6H). The element in its entirety closely adheres to the narial margins but does not contact the adjacent elements. The absence of a contact with the premaxilla is the result of postmortem displacement, as there is a shallow concavity on the internal surface of the premaxilla to accommodate the septomaxilla (Figs. 6C and 6D). This surface is not exposed in any specimen of *Hapsidopareion*.

The lacrimal is a subtriangular element that contributes to the anterior and ventral margins of the orbit (Figs. 2, 4, 6I and 6J). In these specimens, it differs from the more rectangular contour reconstructed by Bolt & Rieppel (2009) in having a relatively slender anterior process that tapers from the posterior portion. Consequently, this suggests that the anteriormost portion of the snout is not as tall as previously reconstructed. The shape of the lacrimal is more like that of *Saxonerpeton* (Carroll & Gaskill, 1978) than that of *Hapsidopareion* (Daly, 1973). In the latter, the anterior tapering is minimal, resulting in a greater contribution to the posterior narial margin and a shorter distance between the naris and the orbit. The internal surface of the anterior orbital margin is reinforced by a ventromedial process of the lacrimal that extends ventral to the paired foramina of the nasolacrimal duct. This surface is in turn buttressed anteriorly by a flange of the prefrontal. In OMNH 73718, the lacrimal appears to have sutured to the palatine. The presence of paired foramina for the nasolacrimal duct (Figs. 6I and 6J) differs from the condition reported in the holotype (Bolt & Rieppel, 2009). It is, however, seen in the co-occurring ostodolepid *Nannaroter*, which features a third foramen interpreted to be for the facial nerve (Anderson, Scott & Reisz, 2009), and in some recumbirostrans (Szostakiwskyj, Pardo & Anderson, 2015). A single perforation is reported in some taxa (e.g., *Asaphestera*, *Pantylus*, *Cardiocephalus*), but expression of the nasolacrimal duct is unreported in a number of other taxa (Carroll & Gaskill, 1978). There is no evidence for a foramen for the facial nerve, which is seen in *Nannaroter*, although several tiny pits are found medial to the nasolacrimal duct in the new specimens of *Llistrofus*. The perceived difference from the holotype (Bolt & Rieppel, 2009) may be the product of damage to the thin septum dividing the two foramina seen in OMNH 79031 or poor visibility of this region.

The prefrontal is a rectangular element sutured to the frontal medially and anteroventrally to the lacrimal by a robust ventral flange (Figs. 2–4). Small pitting can be found at the posterolateral margin near the orbit in both specimens. The posterior process of the prefrontal is reduced in comparison to those in *Saxonerpeton* and *Hapsidopareion*, in which it contacts the postfrontal to exclude the frontal from the orbital margin. Bolt & Rieppel (2009) were uncertain about the right prefrontal of the holotype, which appears to end abruptly posteriorly along the orbital margin in a rounded terminus that does not partially incise posteromedially into the frontal, in contrast to the left prefrontal. The condition of the left prefrontal of the holotype is seen in both new specimens, and therefore the aberrant condition of the right prefrontal of the former is likely the result of its fragmentary nature (Bolt & Rieppel, 2009:fig. 1).

**Dermal skull roof.** The nasals of the new specimens are comparable to those of *Saxonerpeton* in being square-shaped and in terminating well before the anterior orbital margin (Figs. 2–4). Those of *Hapsidopareion* are more rectangular and terminate approximately at the same longitudinal distance as the anterior margin. Both pairs of nasals in the new specimens are partially divided anteriorly along the shared midline suture (Figs. 3 and 4); this does not appear to be solely the result of taphonomic damage. Since the premaxillae do not contact the nasals in either specimens, it is difficult to conclude whether the dorsal exposure of the premaxillae extended posteromedially to create this division. However, it seems plausible that this could have been accomplished through an alary process, as in *Hapsidopareion* (Daly, 1973; Carroll & Gaskill, 1978) and as we previously noted. The curvature and the posterodorsal tapering of the premaxillae, particularly in OMNH 79031 (Fig. 6D), support this interpretation.

The frontals of OMNH 73718 are incomplete posteriorly, and the left frontal is split in half transversely, with the anterior half dipping ventromedially; the posterior half remains articulated with the postfrontal (Fig. 3). Intraspecific variation may be noted in the relationship between the frontals of *Llistrofus*. In OMNH 73718, the right frontal appears to be medially expanded, while the left frontal appears to be medially incised. The frontals of OMNH 79031 are similarly characterized by a transverse incision of the right frontal into the left frontal. The incision is bracketed by smaller incisions in the opposite direction (from left into right) (Fig. 4). In the holotype, the inverse relationship is observed: a transverse flange of the left frontal incises into the right frontal at the mid-point of the bone (Bolt & Rieppel, 2009). The incising flange is framed by two flanges that similarly incise in the opposite direction across the midline. This articulation is also noted in *Hapsidopareion* (Daly, 1973). The absence of a prefrontal–postfrontal contact in all specimens allows the frontals to contribute to the dorsomedial margin of the orbit (Bolt & Rieppel, 2009). The frontals of *Saxonerpeton* and *Llistrofus* are narrower than those of *Hapsidopareion*, leading to an increased dorsal exposure of the orbits in the former two. Additionally, the frontals of *Saxonerpeton* and *Llistrofus* originate well anterior of the orbits and terminate slightly posterior to them (Carroll & Gaskill, 1978); those of *Hapsidopareion* originate slightly anterior to the orbits and terminate well posterior to them (Daly, 1973). As a result, the prefrontal region of the latter is subequal in length to the postfrontal region, rather than being notably shorter as in *Llistrofus*. The presence of a ventral flange toward the anterodorsal edge of the orbit was not recognized in the holotype (Bolt & Rieppel, 2009) and was only recognized in the scan data in OMNH 79031. This flange is more transversely thickened and not ventrally extensive, and it contacts the orbitosphenoid in an abutting joint (Fig. 5C). The condition of *Hapsidopareion* and *Saxonerpeton* is unclear.

The parietals are large, subrectangular elements that enclose the pineal foramen (Figs. 3 and 4). Figures and reconstructions of the holotype (Carroll & Gaskill, 1978; Bolt & Rieppel, 2009) show marked differences between the left and the right parietals with respect to their posterior portions. In the former, the posterolateral extent reaches nearly to the occipital flange of the postparietal, while in the latter it is much more truncated due to an expansion of the right postparietal. In OMNH 79031, the morphology and posterior extent of the two parietals appear more symmetrical where preserved. The parietals of *Llistrofus*, like the frontals, are longer anteroposteriorly than in *Saxonerpeton*, which in turn has longer parietals than *Hapsidopareion* (Daly, 1973; Carroll & Gaskill, 1978). As with the frontals, the left parietal of OMNH 79031 incises into the right parietal. The outline of the pineal foramen is marked by a thickened ridge that elevates the opening above the plane of the other roofing elements, and a corresponding thickening is also present on the ventral surface. The pineal foramen is more posteriorly situated than in *Hapsidopareion* (Daly, 1973).

The postparietals are subtriangular in outline, being expanded medially with a slender lateral process that meets the tabular (Fig. 4). There is no evidence of the postparietals extending to the posterolateral skull corner, as suggested by Carroll & Gaskill (1978). The posteromedial surface is slightly dorsally convex where it overlaps the anterior portion of the supraoccipital. The postparietals of OMNH 79031 notably taper laterally, producing the subtriangular profile, in comparison to the holotype in which a more minor degree of tapering produces more rectangular postparietals (Bolt & Rieppel, 2009). The latter condition more closely resembles that seen in other “microsaurs” (including *Hapsidopareion* and *Saxonerpeton*). The contact with the parietals is more complex in the holotype, with several rounded interdigitations (Bolt & Rieppel, 2009), in contrast to the straight edges of OMNH 79031 (Fig. 4). In the holotype, the left postparietal incises into the right postparietal at the posterior margin in a similar fashion to the frontals (Carroll & Gaskill, 1978; Bolt & Rieppel, 2009). In OMNH 79031, as with the frontals, the contact between the postparietals in the latter shows the inverse condition (reflecting intraspecific variation), with the right postparietal incising into the left postparietal. A similar contact is illustrated for *Hapsidopareion* but appears absent in the postparietals of *Saxonerpeton* (Carroll & Gaskill, 1978). A portion of the occipital flange descends ventrally from the right postparietal and contacts that of the tabular laterally.

Carroll & Gaskill (1978) noted a palpebral bone along the left dorsal orbital margin of *Llistrofus*. Bolt & Rieppel (2009) tentatively identified fragments of a palpebral cup, although they argue that the reconstructed crescentic shape of previous authors was not supported. A few small, flat, slightly curved fragments are found near the dorsal margin of the left orbit in OMNH 79031 (Figs. 2 and 4). They are somewhat similar to those reported in the right orbit of the holotype and collectively form somewhat of a crescentic shape, as reported by Carroll & Gaskill (1978) but disputed by Bolt & Rieppel (2009). However, they could very well be unrelated fragments, especially given the mixed taxonomy and disarticulation of many elements within the block. Their relative position to the orbit is the strongest evidence for an identification as palpebral bones. Palpebral cups are described in *Hapsidopareion* and not in *Saxonerpeton* (Daly, 1973; Carroll & Gaskill, 1978), although Bolt & Rieppel (2009:482) stated that no palpebral cups are found in *Hapsidopareion*. The loose articulation of these ossifications and the low number of specimens mean that taphonomic loss cannot be ruled out; a small minority of the dozens of specimens of *Microbrachis* preserve such ossifications, for example (Olori, 2015).

**Temporal region.** The jugal contributes to the ventral and posterior margins of the orbit (Figs. 2–4). It is sutured to the postorbital dorsally, to the maxilla ventrally, and likely to the lacrimal anteriorly. The contact with the lacrimal is not preserved in any specimen of *Llistrofus*, but this relationship is found in *Hapsidopareion*, and the processes of the two elements extend along the ventral orbital margin and approach each other very closely in all specimens of *Llistrofus* (Bolt & Rieppel, 2009; Figs. 2 and 3). Its ventral surface is slightly convex to meet the maxilla, and it thins posterior to the terminus of the maxilla. Its dorsolateral surface is slightly excavated for the dorsal overlap of the postorbital. A reduction in the posterior extent of the jugal in hapsidopareiids accompanies the temporal emargination. The posteroventral portion is expanded ventrally so as to descend below the plane of the ventral margin of the maxilla, a rare feature in “microsaurs” that is otherwise seen to a lesser degree in *Asaphestera* (as reasonably reconstructed by Carroll & Gaskill, 1978) and more pronouncedly in *Pantylus* (Romer, 1969). The morphology of this process is variable among hapsidopareiids, being more sharply convex posteriorly in *Hapsidopareion* (Daly, 1973) and more rectangular in *Llistrofus*.

The postfrontal is subrectangular and has a thin, subtriangular process that extends ventrolaterally along the posterior orbital margin of the orbit, where it contacts the postorbital in a shallow dorsal notch in the latter. This process is slightly shorter in *Llistrofus* than in *Hapsidopareion* (Bolt & Rieppel, 2009). The postfrontals of the new specimens differ from the holotype only in having a more rounded posterior termination than that reconstructed by Bolt & Rieppel (2009). The left postfrontal of OMNH 79031 has a well-exposed sutural surface area for the dislodged postorbital. In *Llistrofus*, the postfrontal is excluded from the temporal emargination by the postorbital and the tabular, in contrast to *Hapsidopareion*, where there appears to be a small posterior contribution (the result of a separated postorbital and tabular) (Daly, 1973). However, it is difficult to discern whether this is the actual condition in the latter because of damage to this area (Carroll & Gaskill, 1978:figs. 13A–B, 14G).

The postorbital is a relatively slender element that contributes to the posterior margin of the orbit where it sutures to the jugal ventrally and to the postfrontal dorsally, to the anterodorsal margin of the temporal emargination, and to the tabular posteriorly (Figs. 3 and 4). The ventral process overlaps the dorsal process of the jugal anteriorly in the holotype and in OMNH 79031 (Figs. 3 and 4). The element curves slightly upward posterodorsally into a predominantly dorsal exposure where it contacts the tabular to exclude the postfrontal from the emargination, in contrast to *Hapsidopareion* (Daly, 1973; Carroll & Gaskill, 1978). As a result, the postorbital–postfrontal suture of *Llistrofus* is a continuous horizontal contact, in contrast to the sharply curved contact of *Hapsidopareion* that is formed by the postorbital prominently incising into the ventral region of the postfrontal in lateral profile (e.g., Daly, 1973:fig. 15; Carroll & Gaskill, 1978:fig. 13A–B).

The tabular is a large, rectangular element with a lobe-shaped anteromedial process that lies adjacent to the posterolateral process of the postfrontal. The anteromedial process of the tabular partially separates the parietal and the postfrontal along their posterior sutural contact (Fig. 4). The tabular has a narrow contact with the postorbital laterally to form the dorsal margin of the emargination. The sutural contact between the tabular and the parietal is sharply angled at its posterolateral termination. Posterior to this junction, the tabular descends ventrally into a broad occipital flange that is continuous with that of the postparietal.

The squamosal is slightly convex on the lateral surface (Figs. 3 and 4). A fragmentary element identified as the partial right squamosal of OMNH 79031 overlies the frontal and the postfrontal near the right orbit (Fig. 4). It lacks the dorsal process that would underlie one of the roofing elements. The squamosal is nearly straight except for the medially inclined dorsal subtemporal flange, which is presumed to have underlain the ventral surface of the tabular. Its ventral edge forms an oblique suture with the quadratojugal. The squamosal would have descended posteroventrally from the roof to posteriorly frame the temporal emargination. However, the element is not articulated in any specimen of *Llistrofus*, and the ventral surface of the tabular is smooth in this area, making it difficult to determine the precise vertical and lateral angles of orientation or its position relative to the tabular and the postparietal. However, if it is accepted that the element has merely been splayed laterally postmortem (particularly in the more complete OMNH 79031), it appears that it would have been more posteriorly positioned, as reconstructed by Carroll & Gaskill (1978) and in contrast to *Hapsidopareion* in which some specimens possess a squamosal that is closer to the mid-length of the tabular (Carroll & Gaskill, 1978:fig. 13C). The angle between the subtemporal flange and the body of the squamosal is the same as in the holotype (approximated as 140°), which indicates that the element would have flared ventrolaterally, as interpreted by Bolt & Rieppel (2009). The vertical orientation of the squamosal is slightly posteroventrally angled (Bolt & Rieppel, 2009), as in *Hapsidopareion* (Daly, 1973; Carroll & Gaskill, 1978).

The quadratojugal is a small element, triangular in lateral profile, with one process extending dorsally and another extending anteroventrally (Fig. 4). The posterior margin of the dorsal process jointly frames a small foramen with the squamosal (Fig. 4). This foramen passes between the two elements and is also seen in the holotype (Bolt & Rieppel, 2009). The anteroventral process contributes to the posteroventral corner of the temporal emargination. The dorsal process contacts the squamosal at an oblique angle while the ventral contact with the quadrate is mostly horizontal. Bolt & Rieppel (2009) suggested that the posterior portion of the quadratojugal was clasped between the squamosal and the quadrate, which is confirmed in OMNH 79031. The tip of the dorsal process forms a point, not the squared-off end identified in the holotype (Bolt & Rieppel, 2009), which may be a taphonomic artifact in the latter. The anteroventral process also tapers and has a straight ventral margin, contrary to the markedly convex margin identified in the holotype (Bolt & Rieppel, 2009). The cause of this disparity is unclear, as the ventral region appears to be undamaged in both the holotype and in OMNH 79031. The quadratojugal is unknown in *Hapsidopareion*, but this may be due to reduced preservation potential associated with its small size.

The quadrate is a subtriangular element (Figs. 2 and 4; Fig. S1). In lateral profile, the dorsal process is broadly expanded mediolaterally. In posteromedial view, the dorsal process is divided into two discrete anteroposteriorly separated processes that project dorsally at the same angle as the squamosal (Fig. S1). These are joined into a single thin, vertical ridge at the anterior edge, as in the holotype (Bolt & Rieppel, 2009), but they flare slightly toward the contact with the squamosal, accentuating the separation (Fig. S1). Presumably, these were for articulating with the quadrate ramus of the pterygoid and the squamosal. The medial surface is slightly concave for contact with the quadrate ramus (Fig. S1). The articular surface comprises well-ossified, differentiated condyles, as in the holotype (Bolt & Rieppel, 2009).

**Ornamentation.** *Llistrofus* has a number of irregularly spaced pits and shallow grooves on the surface, although the dorsal surface is predominantly smooth (Figs. 3 and 4). In several elements, such as the nasal, the frontal, and the postfrontal, a few small pits are located near the center of the element and transition toward radiating grooves. The jugal is the most heavily ornamented element, as it has more pronounced grooves that are bounded by raised ridges near the orbital margin. There is no evidence of lateral line sulci in *Llistrofus*, and there is no ornament on the mandibles. The ornamentation of *Llistrofus* is modest in comparison to rugose forms such as *Pantylus* (Romer, 1969), mature specimens of *Microbrachis* (Olori, 2015), or the co-occurring dissorophoid temnospondyls. Most other “microsaurs” fall somewhere between the absence of ornamentation seen in *Hapsidopareion* and the developed ornamentation of *Pantylus*. That of *Llistrofus* is comparable to *Sparodus* and *Tuditanus* according to Carroll & Gaskill (1978), while the description of sculpturing in *Hyloplesion* and immature *Microbrachis* by Olori (2015) is also comparable to that which we observed. It is more pronounced than in *Saxonerpeton* and *Hapsidopareion*, both of which are described as having nearly smooth skulls (Daly, 1973; Carroll & Gaskill, 1978).

**Sutural relationships.** As noted in the earlier description, the surficial expression of the sutures is relatively simple, with a few simple interdigitations (e.g., the median interdigitation between the frontals and between the parietals). Additional information is revealed through the tomographic data. Most of the contacts are marked by thin, underplating flanges that shallowly angle (Fig. 5), with most flanges being directed anteroposteriorly. The median roofing elements (frontal, parietal, postparietal) send underlapping flanges anteriorly. This is also true of the more lateral elements (e.g., tabular beneath the postfrontal). Lateral underplating flanges are seen in the parietal (under the tabular and the postfrontal), in the postfrontal (under the frontal along the orbital margin), and in the nasal (under the prefrontal). The contact between the premaxillae, that between the nasals, the reinforced antorbital region (prefrontal–lacrimal), and the postorbital–-postfrontal contact are abutting joints. Contact between the frontal and the orbitosphenoid is also an abutting joint. Some inferred abutting joints have been loosely disarticulated (e.g., lacrimal–maxilla, jugal–maxilla, squamosal–tabular). The only evidence of a more complex suture is a tongue-and-groove joint between the frontal and the posterior process of the prefrontal along the orbital margin.

**Figure 7:**
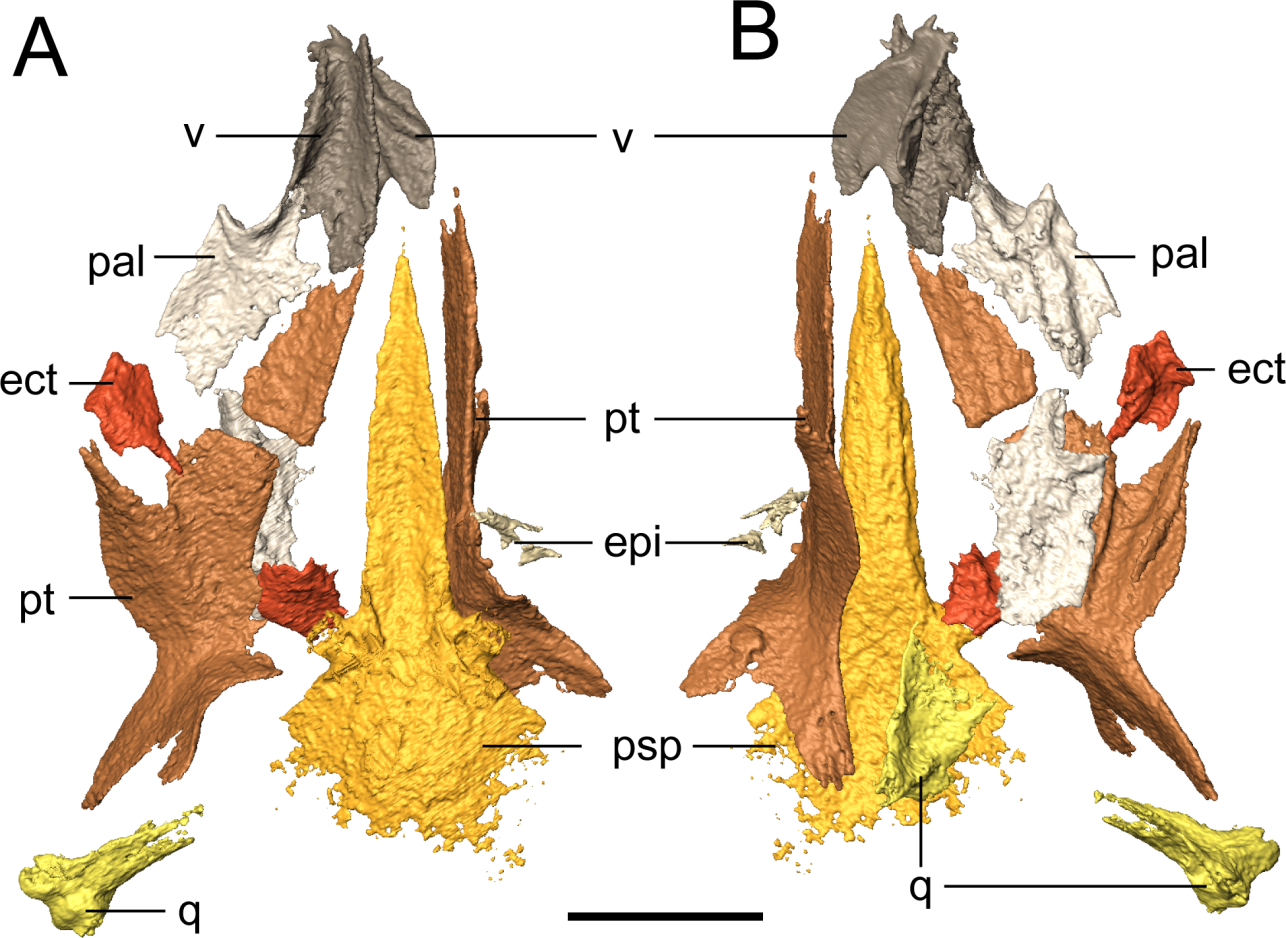
Selected profiles of the palate of referred specimen of *Llistrofus pricei* (OMNH 79031). (A) Dorsal profile; (B) ventral profile. The putative left epipterygoid is excluded for scaling purposes given its dislocation relative to the rest of the palate. Abbreviations: ect, ectopterygoid; epi, epipterygoid; pal, palatine; psp, parasphenoid; pt, pterygoid; q, quadrate; v, vomer. Scale bar equal to four mm.

**Palate.** The parasphenoid is formed by the broad, trapezoidal basal plate and the elongate cultriform process (Fig. 7). Slight weathering has occurred to the posterior margin of the basal plate, but the margin is inferred to have been convex, as with the holotype (Bolt & Rieppel, 2009). The basipterygoid processes are well-defined. One notable difference from the holotype is that there is no separation of the cultriform process from the basal plate, one of the few diagnostic features used by Bolt & Rieppel (2009) to differentiate *Llistrofus* from *Hapsidopareion*. This is otherwise unreported in other “microsaurs” and is interpreted here as a taphonomic artifact. A coarse texture interpreted as a covering of denticles is discernible from the scan data. The holotype preserves the typical denticle field found on the basal plate in many “microsaurs” (Bolt & Rieppel, 2009). The cultriform process is of a typical “microsaurian” morphology, being broad and parallel-sided throughout most of its length and with a dorsally concave surface that deepens at the base of the cultriform process, presumably for the hypophyseal fossa.

The pterygoid is a long, complex element that spans much of the length of the palate and that includes a number of rami (Fig. 7). The prominent quadrate and palatine rami are presumed to have framed a deep basicranial recess (Bolt & Rieppel, 2009). Bolt & Rieppel (2009) suggested, based on the size of the recess, that it is the epipterygoids that formed the basipterygoid articulation. However, neither the pterygoids nor the epipterygoids are articulated in any specimen of *Llistrofus*. The palatine ramus is not well-exposed in the holotype, but as revealed in this study, it has a well-developed, anterolaterally directed trough along its ventral surface that is framed by discrete ridges (Fig. 7B); this can also be seen in recumbirostrans (Szostakiwskyj, Pardo & Anderson, 2015). A coarse texture similar to that of the parasphenoid is inferred to represent a denticle field, which is exposed in the holotype. Based on the position of the vomer, which likely contacted the pterygoid posteriorly (as in the holotype), we can conclude that similar to *Saxonerpeton*, the pterygoid was probably separated from the premaxilla. This is in contrast to the condition in *Hapsidopareion* where the two elements contact (Daly, 1973; Carroll & Gaskill, 1978). However, in some specimens of *Hapsidopareion* (Carroll & Gaskill, 1978:28–29, figs. 13A, 14E), the vomers appear to be anterior, not lateral, to the anterior extent of the pterygoid and are much wider than in reconstructions (Daly, 1973:577, fig. 16; Carroll & Gaskill, 1978:28, fig. 13B). This suggests that the pterygoid and premaxilla may have been separated, as in *Llistrofus* and *Saxonerpeton*. Separation of these elements is found in most “microsaurs.” *Microbrachis* was reconstructed by Carroll & Gaskill (1978) as having the condition of *Hapsidopareion*, but this has since been corrected by Olori (2015), who noted the more typical separated condition.

The only evidence of an epipterygoid is a fragmentary element anterior to the right stapes that is separated from the rest of the braincase by the dislodged right pterygoid (Figs. 1 and 7). It comprises a well-defined cylindrical shaft and what appears to be a highly fragmentary ventral expansion. The preserved portion is consistent with that of the epipterygoid in taxa in which it is well-preserved, such as *Carrolla, Rhynchonkos*, *Huskerpeton*, and *Brachydectes* (Maddin, Olori & Anderson, 2011; Huttenlocker et al., 2013; Szostakiwskyj, Pardo & Anderson, 2015; Pardo & Anderson, 2016). When viewed in palatal view (Fig. 7), it is not significantly displaced from the basipterygoid articulation, with which it could have articulated, being only dorsally displaced and separated by the dislodged pterygoid. A second element with a similar shaft that appears to be expanded at one end is found next to the atlas-axis complex and is tentatively identified as the other epipterygoid (Fig. 1F; Movie S1).

The vomer is a subrectangular element that sutures posteriorly to the pterygoid and posterolaterally to the palatine (Fig. 7). The left vomer is dislodged to directly overlie the right vomer (Fig. 7). We agree with Bolt & Rieppel (2009) that the restricted anterior extent of the pterygoids suggests that the vomers shared a medial contact for much of their length. The smooth, straight medial margin also supports this inference, but the precise extent cannot be defined in OMNH 79031. The vomer is rectangular and longer anteroposteriorly. It forms nearly the entirely of the medial margin of the internal naris and is assumed to have sutured to the pterygoid directly posteriorly, rather than overlapping the anterior process of the pterygoid as was suggested in the holotype by Bolt & Rieppel (2009). The left vomer of the holotype appears to be dislodged into a different plane from that of the pterygoid (Bolt & Rieppel, 2009:fig. 3) and into an artificial overlapping relationship. Along its medial margin, the element curves dorsally to form a prominent flange that extends posteriorly toward the pterygoid, gradually rises to form a convex margin, and then terminates just before the anterior margin of the vomer; this feature is also seen in the holotype (Bolt & Rieppel, 2009). An ascending flange is also found around the edge of the choana, as with the holotype (Bolt & Rieppel, 2009). The element probably bore denticles (found in the holotype), but the texture of the element is not well resolved in the scan data. The vomer of *Llistrofus* is more like that of *Saxonerpeton* in being much wider than in *Hapsidopareion* (Daly, 1973; Carroll & Gaskill, 1978). In *Hapsidopareion*, a slender vomer could have permitted a greater anterior reach of the pterygoids, but as noted above, there are some discrepancies between the specimen illustrations and the reconstructions (Daly, 1973; Carroll & Gaskill, 1978).

The rectangular palatine contacts the vomer anteromedially and the pterygoid posterolaterally (Fig. 7). It probably sutured to the maxilla and contributes to the posterior and medial margins of the internal naris (Fig. 7). The posterior margin of the choana is formed by a dorsal flange that is continuous with that of the vomer and that continues to the posterolateral margin. In dorsal profile, this produces a longitudinal trough posterior to the choana. The lateral margin is slightly thicker than the medial one, presumably to contact the maxilla. Ventrally, a semicircular tooth-bearing ridge is positioned just posterior to the choana and is continuous with a longitudinal ridge that is more laterally positioned, producing a contour in the shape of an “open-top 4.” In the holotype, this ridge bears several small teeth, and in OMNH 79031, a minimum of eight tooth positions can be tentatively identified on the posterior portion of the ridge. It is impossible to compare either the total count or the morphology of these teeth due to their small size at the scanned resolution. The six teeth found on this ridge in *Hapsidopareion* were of comparable size to the marginal dentition (Carroll & Gaskill, 1978:28), in contrast to the notably smaller teeth seen in the holotype of *Llistrofus* and in OMNH 79031. The element does not appear to bear the texture representing denticles that was found on other bones, and denticles are absent in the holotype (Bolt & Rieppel, 2009). The right palatine is complete but now lies ventral to the left side of the palate and is exposed in dorsal profile when the specimen is viewed in palatal profile.

The rectangular ectopterygoids are disarticulated but presumably sutured to the maxilla laterally, to the palatine anteriorly, and to the pterygoid medially (Fig. 7). The element has a laterally curving trough on the ventral surface, bounded by a thin ridge. The ridge is laterally continuous with the lateral margin of the palatine and that is ventrally convex, with a posterodorsal curve. It appears that there is a small anteromedial patch of teeth, more resolved than the inferred denticulate texture but smaller than the marginal teeth that is continuous with those on the palatine. These probably correspond with the partially exposed teeth that were identified as possibly pertaining to the ectopterygoid in the holotype (Bolt & Rieppel, 2009). A posteromedially angled process is also present. The ectopterygoid is proportionately small in *Saxonerpeton* and *Hapsidopareion* (Daly, 1973; Carroll & Gaskill, 1978) compared to other “microsaurs (but see *Carrolla*; Maddin, Olori & Anderson, 2011 for another example) in which it is usually of a subequal size to the palatine (Carroll & Gaskill, 1978; Szostakiwskyj, Pardo & Anderson, 2015). The ectopterygoid of *Aletrimyti* is poorly ossified but may be of a comparable relative size (Szostakiwskyj, Pardo & Anderson, 2015). Many gymnarthrids have been reconstructed as having similarly small ectopterygoids (e.g., Carroll & Gaskill, 1978: fig. 109). However, the vast majority of relatively complete gymnarthrid specimens (e.g., the types of *Euryodus primus* and *E. dalyae*) possess articulated mandibles that obscure the lateral extents of the palate (Gregory, Peabody & Price, 1956) such that the interpretation of the proportions of the elements is often speculative.

**Figure 8:**
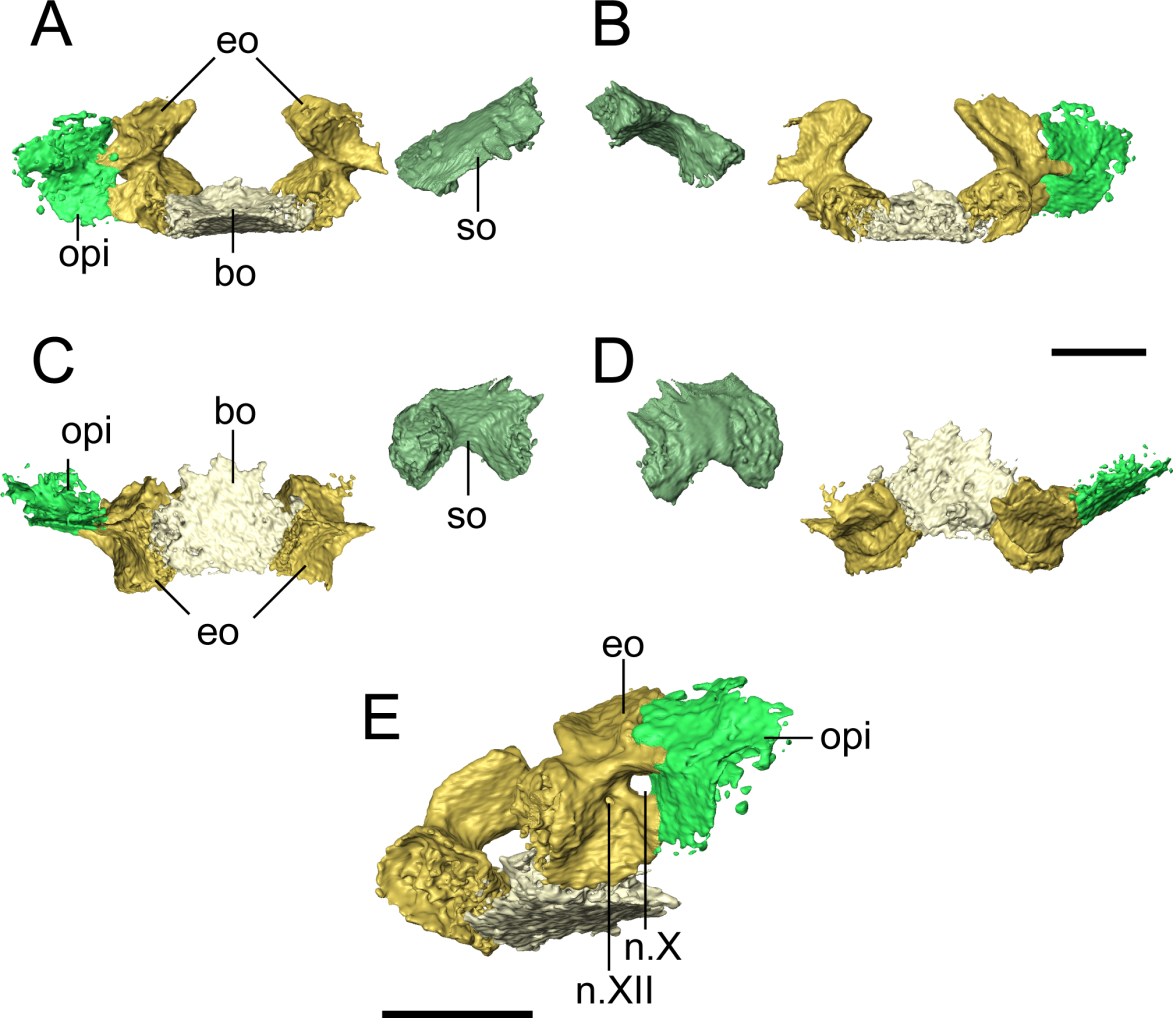
Selected profiles of the occiput of referred specimen of *Llistrofus pricei* (OMNH 79031). Profiles are oriented relative to the basioccipital-exoccipital complex. (A) Anterior profile; (B) posterior profile; (C) ventral profile; (D) dorsal profile; (E) right posteroventrolateral profile showing nerve foramina. Abbreviations: bo, basioccipital; eo, exoccipital; n.X, metotic foramen for the vague nerve and the jugular vein; n.XII, hypoglossal nerve foramen; opi, opisthotic; so, supraoccipital. Scale bars equal to four mm.

**Occiput.** The supraoccipital is an unpaired median element that extends posteroventrally from the posterior skull table, being overlain anteriorly by the postparietals (Figs. 1, 2, 4 and 8). In occipital view, the element is wide and mostly flat, with a dorsally convex ventral margin that frames the foramen magnum dorsally. Large posterolaterally directed triangular facets would have articulated with the exoccipitals (Fig. 8). The general morphology is similar to that of previously studied recumbirostrans in which it is a distinct element (Huttenlocker et al., 2013; Szostakiwskyj, Pardo & Anderson, 2015; Pardo & Anderson, 2016), but it lacks a dorsomedial protuberance (median ascending process) that is commonly found in these taxa. Anteriorly extensive processes (lateral ascending processes) that brace the ossification to the lateral neural wall and that are often found in recumbirostrans are also absent. As a result, the supraoccipital is anteriorly restricted, being most comparable in this regard to *Dvellecanus* and to *Brachydectes* to a lesser extent (Szostakiwskyj, Pardo & Anderson, 2015; Pardo & Anderson, 2016). The absence of those processes is also noted in *Quasicaecilia* in which the synotic tectum is co-ossified into the otoccipital complex (Pardo, Szostakiwskyj & Anderson, 2015). The supraoccipital is not identified in *Carrolla* but was suggested by Maddin, Olori & Anderson (2011) to be similarly co-ossified in the posterior braincase complex. The supraoccipital underplates the postparietals anteriorly for a short distance, terminating posterior to the parietal. Reconstructions and descriptions of the element in *Hapsidopareion* are too generic to be compared to that of *Llistrofus*, and it is not figured in *Saxonerpeton* (Carroll & Gaskill, 1978:figs. 13,17–21).

The basioccipital is a trapezoidal element that widens for a short distance anteriorly before tapering again (Fig. 8). It is about as long as it is wide. It is not as well-ossified as some other elements of the occiput, so the precise contours are not well-defined. The posterior margin appears to have been concave, as in the holotype (Bolt & Rieppel, 2009), to accommodate the atlantal odontoid. In the holotype, Bolt & Rieppel (2009) noted a distinct sheet of bone between the parasphenoid and the exoccipital condyles that would cover the basioccipital and the exoccipitals in ventral profile. Because of the dislodging of the occiput from the rest of the skull, such a sheet could not be distinguished from various other flat fragments of a generic morphology that are preserved within the block. The exoccipitals are robust, with dorsal processes that frame the foramen magnum laterally and that would have contacted the supraoccipital (Fig. 8). The occipital condyles project posteromedially, and the ends have a flat, unfinished bone surface that does not appear to be greatly altered by weathering. The exoccipitals remain distinct elements, unlike in some generalized recumbirostrans (Huttenlocker et al., 2013; Szostakiwskyj, Pardo & Anderson, 2015) and in brachystelechids (Maddin, Olori & Anderson, 2011; Pardo, Szostakiwskyj & Anderson, 2015) in which they partly or completed co-ossify with other occipital or neurocranial elements The metotic foramen for both the vagus nerve (X) and the jugular vein is clearly defined by the right exoccipital and the right opisthotic of OMNH 79031 in which both foramina are present (Fig. 8E), and the posterior margin of the foramen is present in the left exoccipital. The shared contribution by both the exoccipital and the opisthotic is widely found in recumbirostrans (Szostakiwskyj, Pardo & Anderson, 2015; Pardo & Anderson, 2016); often CT data has provided a clearer picture to correct early workers interpretations of a contribution by only the exoccipital. A recess between the exoccipital and the opisthotic was interpreted as this foramen in the holotype of *Llistrofus* by Bolt & Rieppel (2009). In OMNH 79031, a small foramen tentatively identified as that for the hypoglossal nerve (XII) is visible slightly posteroventromedial to the metotic foramen that perforates near the base of the dorsal process of the exoccipital and exists ventrolaterally (Fig. 8E). A foramen in the same position in the holotype was suggested as a possible hypoglossal nerve foramen by Bolt & Rieppel (2009). In some recumbirostrans (e.g., *Aletrimyti*, *Rhynchonkos*), the hypoglossal nerve foramen is more ventrally situated, being framed below by the basioccipital (Szostakiwskyj, Pardo & Anderson, 2015), whereas in others (e.g., *Carrolla*, *Brachydectes*), it appears to be entirely self-enclosed within the exoccipital as in OMNH 79031 (Maddin, Olori & Anderson, 2011; Pardo & Anderson, 2016).

**Figure 9:**
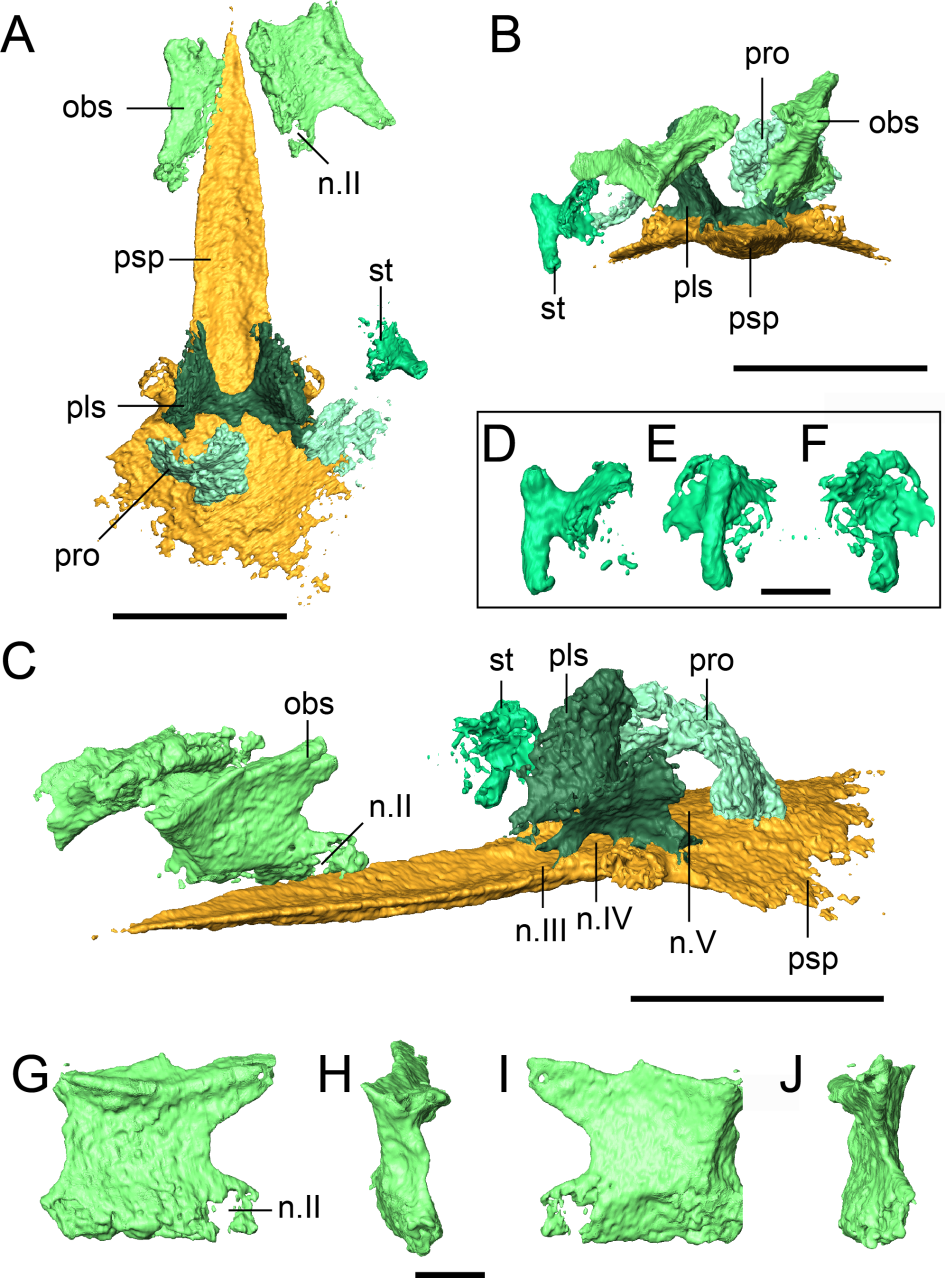
Selected profiles of the neurocranium and otic capsule of referred specimen of *Llistrofus pricei* (OMNH 79031). (A) Neurocranium and otic capsule (dislodged right opisthotic excluded for spacing) in dorsal profile; (B) the same in anterior profile; (C) the same in anterolateral profile; (D) isolated right stapes in anterior profile; (E) the same in lateral profile; (F) the same in medial profile; (G) isolated right orbitosphenoid in medial profile; (H) the same in anterior profile; (I) the same in lateral profile; (J) the same in posterior profile. Dislodged right opisthotic excluded. Abbreviations: n.II, optic nerve foramen; n.III, oculomotor nerve foramen; n.IV, trochlear nerve foramen; n.V, fenestra prootica; obs, orbitosphenoid; pls, pleurosphenoid; pro, prootic; psp, parasphenoid; st, stapes. Scale bars equal to four mm (A–C); one mm (D–J).

**Neurocranium.** At the base of the cultriform process are two ascending flanges that are sutured to the parasphenoid and that meet medially dorsal to the parasphenoid, where they frame the hypophyseal fossa posteriorly (Fig. 9). These are identified as the pleurosphenoids and are commonly found in recumbirostrans in a similar configuration, although they are sometimes specified as the dorsal laminae of the basisphenoid (e.g., Maddin, Olori & Anderson, 2011; Huttenlocker et al., 2013). The basisphenoid itself is not ossified in this specimen. The right pleurosphenoid is more complete and artificially contacts the ventral surface of the parietal posterior to the pineal foramen. In lateral profile, a circular opening is partially framed dorsally, ventrally, and posteriorly at the anteroventral corner of the pleurosphenoid that likely represents the foramen for the oculomotor nerve (III) (Fig. 9C). More posteriorly and just anterior to the basipterygoid processes of the parasphenoid is a large foramen interpreted as the foramen for the trochlear nerve (IV) (Fig. 9C). A curved surface along the posterior margin just below the mid-height is probably the fenestra prootica (trigeminal nerve, V) (Fig. 9C), which would be posteriorly bounded by the slightly dislodged prootic.

A paired set of square ossifications of the anterior braincase are present ventral to the frontals and dorsolateral to the cultriform process (Fig. 9). We interpret these as the orbitosphenoids. These ossifications have sometimes been identified as the “sphenethmoid” in other “microsaurs” (Romer, 1969; Daly, 1973; Langston & Olson, 1986; Carroll, 1990; Henrici et al., 2011; Huttenlocker et al., 2013) and in *Llistrofus* (Bolt & Rieppel, 2009), but their homologues are more recently and more frequently identified as the orbitosphenoids (Maddin, Olori & Anderson, 2011; Szostakiwskyj, Pardo & Anderson, 2015; Pardo & Anderson, 2016). They are separated from the cultriform process in OMNH 79031. In the holotype, the orbitosphenoids are laterally splayed, but their ventral margins remain in contact with the process (Bolt & Rieppel, 2009). Each element is square in lateral profile, with subequal height and length, as in the holotype (Figs. 9G–9J). In OMNH 79031, there is a large space separating the orbitosphenoid and the pleurosphenoid, with little postmortem displacement (Figs. 9A and 9C). This is found in many recumbirostrans, although the orbitosphenoid closely approaches the antotic region in some taxa (e.g., *Dvellecanus, Rhynchonkos*) (Szostakiwskyj, Pardo & Anderson, 2015). The left orbitosphenoid contacts a well-developed ventral flange of the frontal. This relationship is seen in recumbirostrans and is formed through an excavation of the dorsal surface of the orbitosphenoid to accommodate the flange. There is no ventral flange of the parietal, either continuous with or distinct from that of the frontal, in OMNH 79031. Such a flange is found in some recumbirostrans where it contacts the dorsal surface of a posteriorly extensive orbitosphenoid (Maddin, Olori & Anderson, 2011; Szostakiwskyj, Pardo & Anderson, 2015). A prominent foramen is present near the posteroventral margin of each element that is interpreted as the foramen for the optic nerve (II) (Figs. 9C and 9G). Maddin, Olori & Anderson (2011) used this landmark to argue that the square anterior ossifications were the orbitosphenoids (the same as in this study) by paralleling them to the orbitosphenoid of batrachians in which the optic foramen is also contained within this ossification (contrary to reptiles). The foramen is fully contained within the orbitosphenoid, in contrast to the oculomotor nerve foramen, which it defines posteriorly in taxa where the orbitosphenoid contacts the pleurosphenoid (e.g., *Rhynchonkos*; Szostakiwskyj, Pardo & Anderson, 2015). A much smaller foramen of uncertain function perforates the posterodorsal region of the orbitosphenoid (Figs. 9G and 9I).

There is also no evidence for additional discrete ossifications of the anterior braincase (presphenoid, mesethmoid) that are typically seen in recumbirostrans (Maddin, Olori & Anderson, 2011; Pardo, Szostakiwskyj & Anderson, 2015; Szostakiwskyj, Pardo & Anderson, 2015; Pardo & Anderson, 2016). The orbitosphenoids are each a single, homogenous element without evidence of suturing or fusion to other elements. Pardo, Szostakiwskyj & Anderson (2015) noted that the cultriform process of *Quasicaecilia* bears a groove for the dorsal articulation with the presphenoid. This is also found in OMNH 79031 (Fig. 8A), but there is no evidence of the presphenoid in this specimen. One possibility is that the presphenoid could have been tightly articulated with the orbitosphenoids and been broken off when these elements were disarticulated. However, there is no evidence from the tomographic data for ventrally positioned fragments of the presphenoid being adhered to either orbitosphenoid, and it seems unlikely that the element would have split perfectly to create symmetry with each orbitosphenoid. The same symmetry and morphology are seen in the holotype (Bolt & Rieppel, 2009). It seems equally unlikely that the presphenoid was replaced by posteromedial extensions of the orbitosphenoids as in *Dvellecanus* (Szostakiwskyj, Pardo & Anderson, 2015).

**Otic capsule.** The stapes is similar to that of *Pantylus*, with a broadly expanded stapedial footplate that is fused to a dorsoventrally oriented stem of a columnar shaft (Romer, 1969; Fig. 9). There is no evidence of a stapedial foramen, a feature found in the stapes of *Pantylus*. This morphology is also markedly different from that of *Hapsidopareion* (Carroll & Gaskill, 1978) in which the stapes is simply a relatively short shaft with a weakly differentiated footplate and shaft. The stapes of various specimens of *Hapsidopareion*, as figured by Carroll & Gaskill (1978), is articulated within the fenestra vestibularis of variably deformed skulls, and as a result, the differences between these taxa may be confounded by partial exposure or taphonomic damage in *Hapsidopareion*.

The opisthotic sutures to the exoccipital and frames the metotic foramen (Fig. 8). A suture is visible along much of the external surface, and the loss of the opisthotic on the left side indicates that they were not partly co-ossified, which is seen in *Huskerpeton* (Huttenlocker et al., 2013), much less as part of the posterior complex of brachystelechids (Maddin, Olori & Anderson, 2011; Pardo, Szostakiwskyj & Anderson, 2015). It is mostly incomplete dorsoventrally and laterally if it is assumed to be part of a larger plate-like structure that contributes to the occiput, as reconstructed by Bolt & Rieppel (2009). It likely contacted descending flanges of the roofing elements (mostly the tabular), but poor exposure and difficulty distinguishing the elements of the otic capsule in the holotype (Bolt & Rieppel, 2009) confounds additional interpretations.

Two curved elements found posterior to the pleurosphenoids and dorsal to the parasphenoid are identified as the prootics in OMNH 79031 (Fig. 9). Neither is particularly well-ossified or characterized by unique morphological features (possibly the result of taphonomic damage), and the right element appears to be more dislodged and rotated. The corresponding left element is concave in anterior profile, bows outward posteriorly and expands slightly dorsomedially. The element is comparable to the recumbirostrans in which it is not co-ossified with other elements in position, general shape, and proportions. However, it should be noted that it is more often concave in posterior view and bows outward anteriorly (Romer, 1969; Szostakiwskyj, Pardo & Anderson, 2015), the opposite of that seen in OMNH 79031 (possibly the result of dislodgement or rotation). There is no clear demarcation of the foramen for the facial nerve (VII), but this is also not always identified in recumbirostrans (Szostakiwskyj, Pardo & Anderson, 2015). The slight dislodgement of the prootics also blurs the outline of the trigeminal nerve foramen (V) (Fig. 9C).

**Figure 10:**
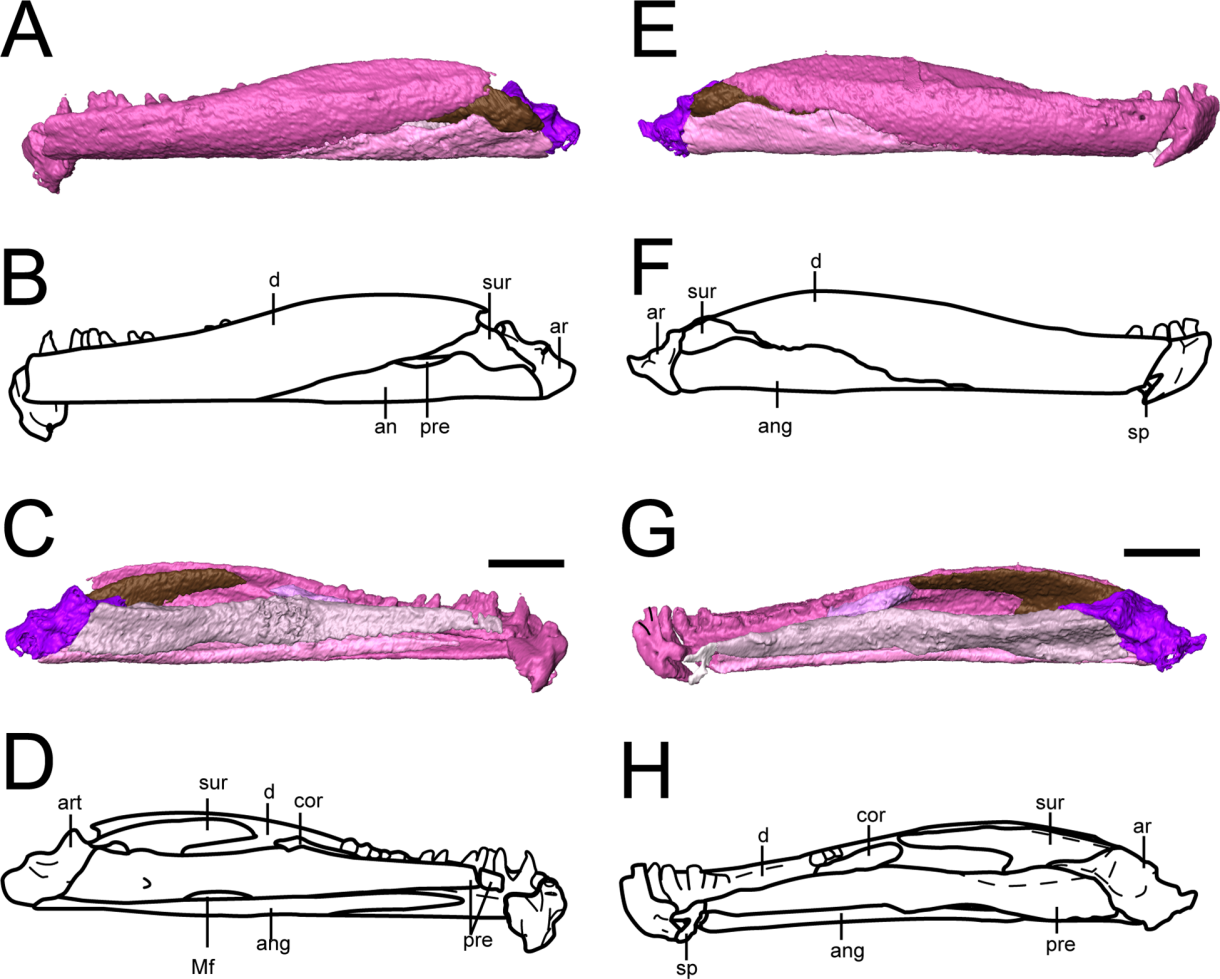
Selected profiles of the mandibles of referred specimen of *Llistrofus pricei* (OMNH 79031). (A) Segmented visualization of the left mandible in lateral profile; (B) outline drawing in the same profile; (C) segmented visualization of the left mandible in medial profile; (D) outline drawing in the same profile; (E) segmented visualization of the right mandible in lateral profile; (F) outline drawing in the same profile; (G) segmented visualization of the right mandible in medial profile; (H) outline drawing in the same profile. Abbreviations: an, angular; ar, articular; cor, coronoid; d, dentary; Mf, Meckelian foramen; pre, prearticular; sp, splenial; sur, surangular. Scale bar equal to two mm.

**Mandible.** The dentary is the largest element of the mandible (Fig. 10). It forms much of the lateral surface, contains the tooth row, and contributes to the low coronoid process, where it sutures to the surangular posteriorly and to the angular posteroventrally. As suggested by Bolt & Rieppel (2009), it extends almost to the posterior end of the mandible. Medially, it sutures to the splenial near the mandibular symphysis, to at least one coronoid at the anterior region of the coronoid process, and to the surangular at the posterior region of the process. The symphysis is broken off in both mandibles of OMNH 79031, which is also seen in the holotype (Bolt & Rieppel, 2009), possibly indicating a region of weakness.

In the holotype, 19 tooth positions were identified, with a total of 25 being estimated (Bolt & Rieppel, 2009). The tooth positions are not as clearly defined in the mandibles as they are in the maxillae or premaxillae of OMNH 79031, but we also identified at least 19 positions in a fully exposed mandible. There is no evidence for an unusually enlarged tooth like that found in the holotype (Bolt & Rieppel, 2009). As with the slightly enlarged tooth that we noted in the maxilla of OMNH 79031, this may reflect intraspecific variation. Replacement is apparently random, and there are only a few replacement sockets (Bolt & Rieppel, 2009).

The splenial is a small element preserved only in the right mandible (Figs. 10G and 10H). It appears slightly dislodged anterior to the prearticular and the angular, and thus the precise orientation of the sutural contacts cannot be discerned, but its general proportions do not appear greatly distorted. This suggests a shorter splenial than previously reconstructed (Bolt & Rieppel, 2009) and a slight contribution to the symphysis. The splenial is smaller than in most other “microsaurs” and does not overlap the prearticular dorsally, a condition shared with *Hapsidopareion* (Carroll & Gaskill, 1978). Whether there was more than one splenial ossification is unclear, as with the holotype (Bolt & Rieppel, 2009).

The prearticular is a long element with a medial exposure that contributes to the medial wall of the adductor chamber (Fig. 10). It sutures to the angular ventrally, with which it frames the Meckelian foramen, to the splenial anteriorly, and to the articular posteriorly. It expands modestly in height posteriorly and is taller than the medial exposure of the angular. As in the holotype (Bolt & Rieppel, 2009), the posterodorsal margin becomes thickened, expanding slightly into the adductor chamber. The suture between the prearticular and the angular was described as “deeply interdigitating” by Bolt & Rieppel (2009), but this area is not preserved in OMNH 73718 and cannot be resolved in OMNH 79031.

The angular is a similarly elongate element that forms the ventral margin of the mandible that also has medial and lateral exposures (Fig. 10). Its anterior extent in both lateral and medial views is greater than that reconstructed by Bolt & Rieppel (2009) (Fig. 10). At least the latter discrepancy may result from uncertainty regarding the precise contact between the angular, dentary, and surangular in the holotype (Bolt & Rieppel, 2009). Its anterior and posterior extents mirror those of the prearticular. In OMNH 73718 (Fig. 3), the articular does not extend as far anteriorly or to the same termination as the prearticular in ventral profile. Whether it has been damaged anteriorly cannot be determined based on the exposed profile and in the absence of a defined splenial. It is possible that the angular and the posterior portion of the splenial are tightly sutured in OMNH 79031 and simply cannot be differentiated at the scanned resolution, as with the right surangular and dentary (details below), producing the artifact of an unusually long angular. As reconstructed here, the angular remains constant in height throughout, being slightly expanded along the mid-length anterior to the Meckelian foramen, which it bounds ventrally. This differs from that of *Hapsidopareion* in which it increases markedly in height posteriorly (Carroll & Gaskill, 1978). The foramen is not reconstructed in *Hapsidopareion* and none is described in the brief discussion of the mandible in *Saxonerpeton* (Daly, 1973; Carroll & Gaskill, 1978). Across “microsaurs,” the presence of the foramen is uncommon; forms in which it is known include *Microbrachis* and *Pantylus* (Romer, 1969; Carroll & Gaskill, 1978). A smaller foramen located posterodorsally to the Meckelian foramen is also present in these specimens, as in the holotype (Bolt & Rieppel, 2009). There is no description of this opening in *Hapsidopareion* and *Saxonerpeton* (Daly, 1973; Carroll & Gaskill, 1978).

Only one coronoid can be confidently identified in these mandibles, being slightly separated from the medial surface of the dentary at the posterior region of the tooth row and anterior to the surangular (Fig. 10). Possibly there was a second coronoid positioned more posteriorly along the coronoid process, but none is apparent in the specimens here. The coronoid process itself is relatively short in comparison to recumbirostrans. The number of coronoids ranges from one to three in “microsaurs,” and the holotype of *Llistrofus* was reconstructed with one (Bolt & Rieppel, 2009). The coronoid is not described for *Hapsidopareion* (Daly, 1973) or *Saxonerpeton* (Carroll & Gaskill, 1978).

The surangular forms the posterior portion of the coronoid process, suturing to the dentary anteriorly, to the angular ventrally, and to the articular posteriorly (Fig. 10). The morphology is in agreement with that of the holotype (Bolt & Rieppel, 2009). The surangular extends anteriorly along the dorsal margin of the coronoid process where it is overlapped by the dentary and extends posteriorly to partially overlap the articular. The lateral exposure is reduced in comparison to other “microsaurs” but is similar to that of *Hapsidopareion* and *Saxonerpeton* in this regard (Carroll & Gaskill, 1978). The resolution of the scan prevented a confident segmentation of the dorsal contact between the right surangular and the dentary (Fig. 10G); the left surangular is detached from the dentary and is thus readily identifiable (Figs. 10C and 10D).

The articular forms the posterior margin of the mandible and forms a well-developed retroarticular process with a broad articular surface (Fig. 10). A retroarticular process is not developed in *Hapsidopareion* (Daly, 1973) and was not previously reported in the holotype of *Llistrofus*. It is better developed in OMNH 79031 (Fig. 10) in which it forms a short but stout process, compared to OMNH 73718 (Fig. 3) in which it tapers to an edge. Because the mandibles of OMNH 79031 are fully encased within the matrix and are completely articulated, differences in the process from OMNH 73718 and the holotype may reflect taphonomic damage. The articular has a restricted lateral profile, although it is greater than that seen in the holotype in which the dentary and the angular obscure it along the ventral margin (Bolt & Rieppel, 2009). A mostly anteroposteriorly oriented ridge divides the lateral and medial portions of the glenoid dorsally, with the medial portion being more elevated anteriorly. A deep pit in the posterior surface of the articular is also noted.

**Figure 11:**
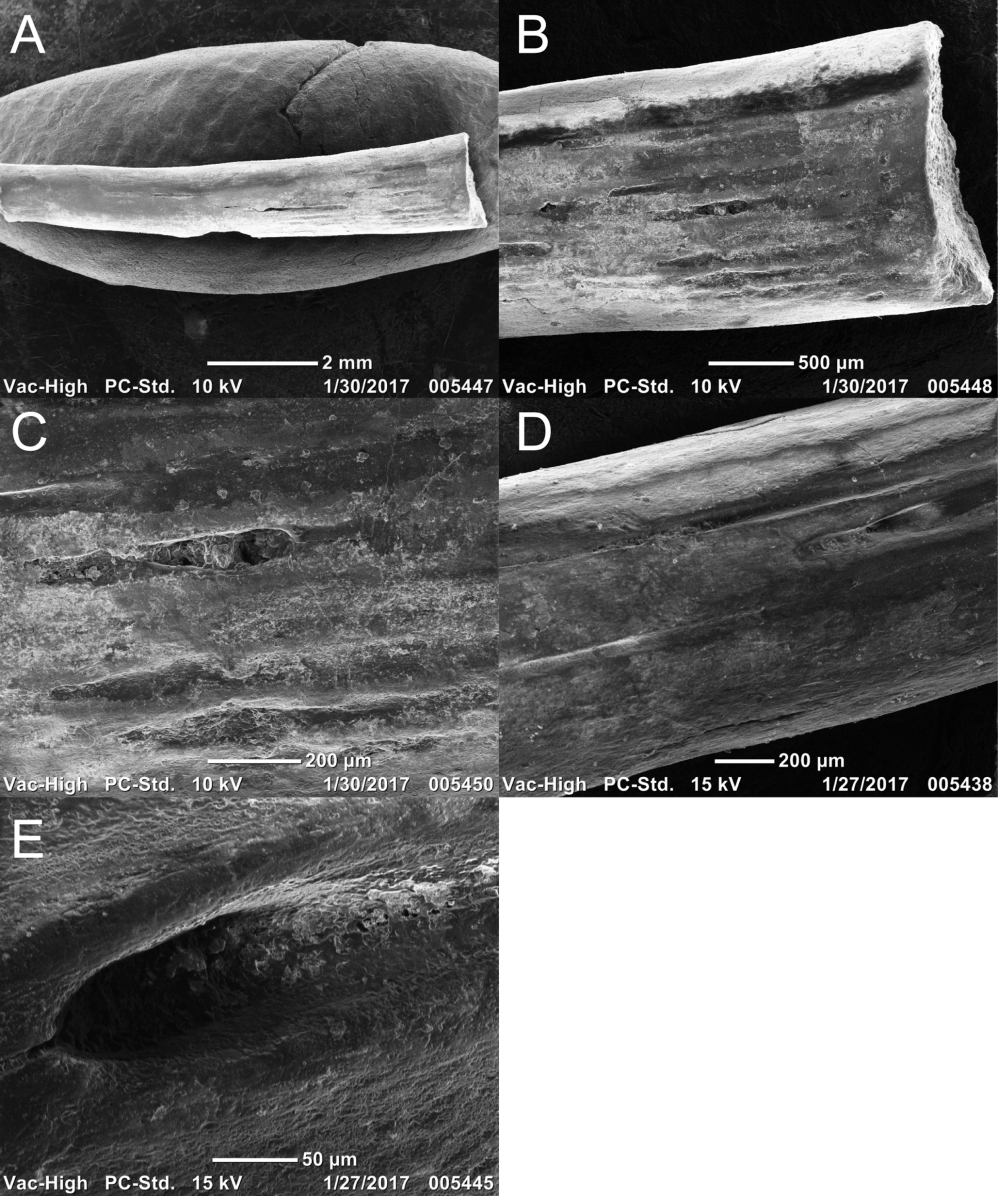
Rib featuring striations of referred specimen of *Llistrofus pricei* (OMNH 79032). (A) SEM image of the entire rib; (B and C) progressive increased magnification on the rib head with striations; (D and E) progressive increased magnification on a striation closer toward the mid-shaft region. Scale bars as listed. Photo credit: Diane Scott.

**Figure 12:**
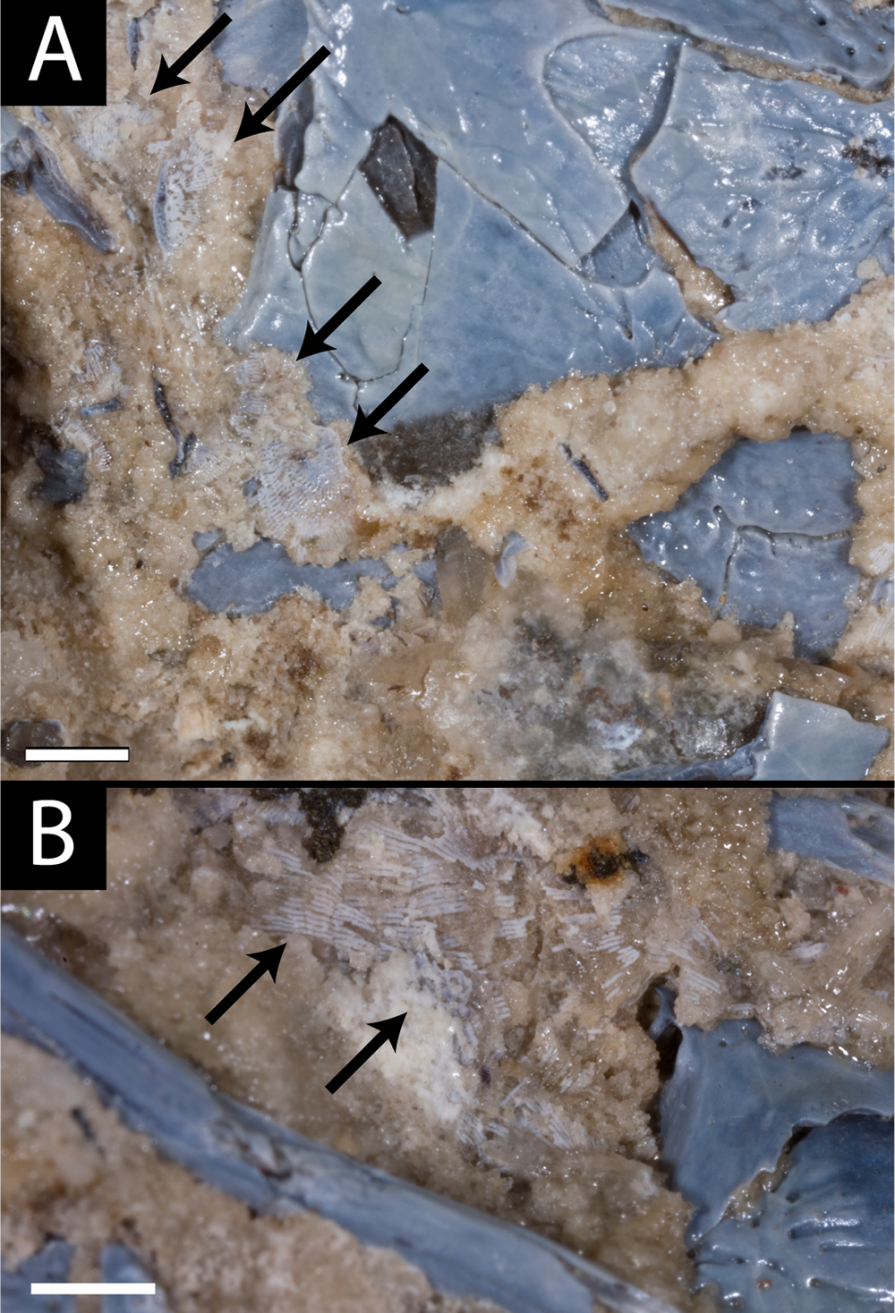
Photographs of the scales of referred specimen of *Llistrofus pricei* (OMNH 79031). (A) Scales (indicated by arrows) located posterior to the pineal foramen along the midline; (B) scales located adjacent to the left orbit. Scale bars equal to one mm. Photo credit: Diane Scott.

**Figure 13:**
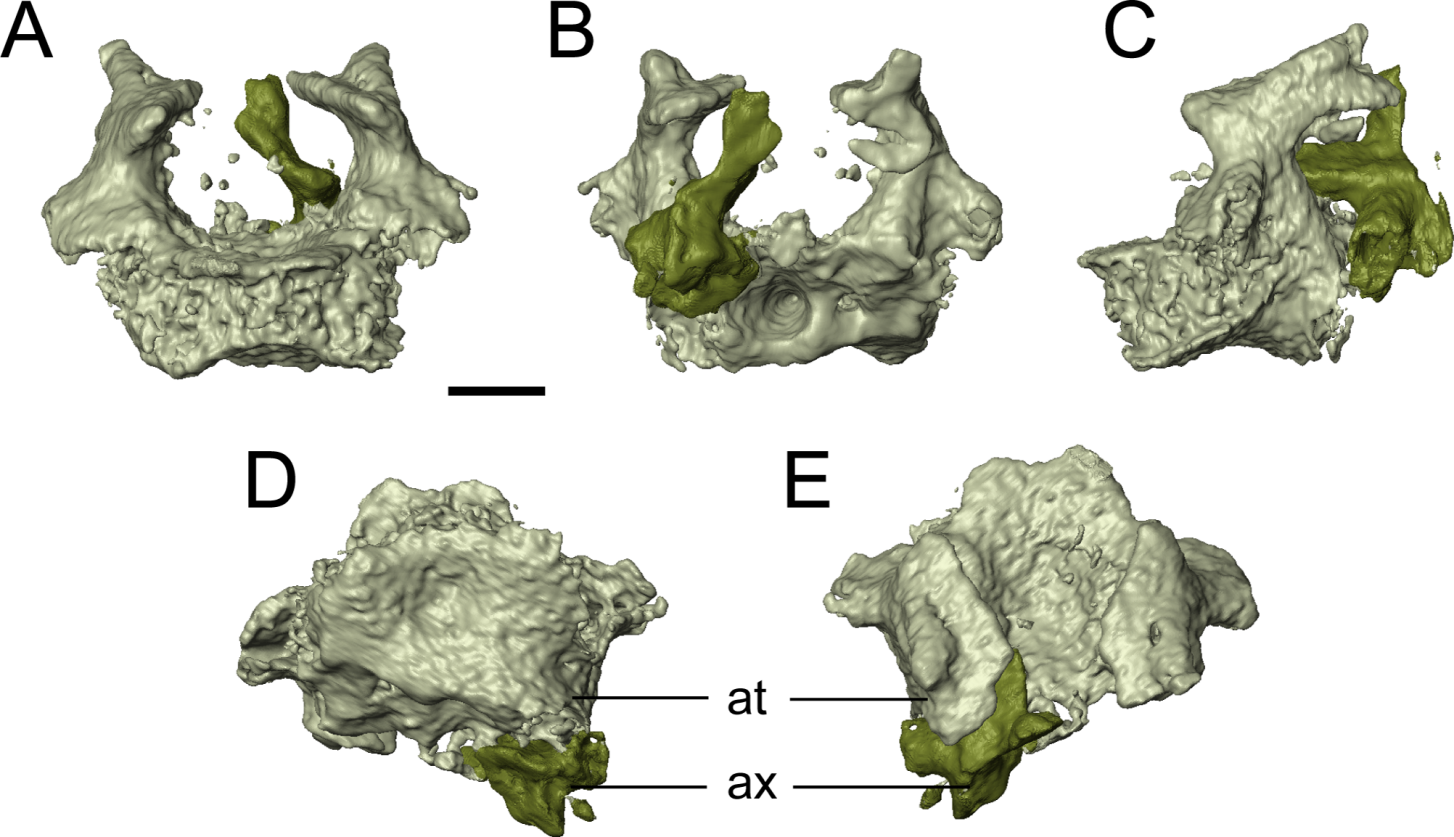
Selected profiles of the atlas-axis complex of referred specimen of *Llistrofus pricei* (OMNH 79031). (A) anterior profile; (B) posterior profile; (C) left lateral profile; (D) ventral profile; (E) dorsal profile. Abbreviations: at, atlas; ax, axis. Scale bar equal to one mm.

**Postcrania.** All of the new postcranial material is part of OMNH 79031 except for OMNH 79032 (isolated rib). Material tentatively associated with the skull includes disarticulated ribs, scales, and vertebrae (Figs. 11–13; Movie S1; Fig. S2). A total of 11 disarticulated ribs are exposed on the block, with an additional four ribs within the block, and there may be an additional two fragmentary ribs. The ribs are double-headed, although the capitulum and the tuberculum are not greatly divided where the proximal ends are preserved. The shaft is cylindrical in cross-section. The ribs of *Llistrofus* are particularly interesting because they are characterized by prominent striations at the ends (Fig. 11), which were figured in the original description by Carroll & Gaskill (1978:fig. 15) but never discussed. All of the exposed ribs of OMNH 79031 bear these characteristic striations, which are best-illustrated in images of OMNH 79032 (Fig. 11). This condition has never been reported or figured in any other “microsaur,” nor has it been found in any of the other Richards Spur tetrapods, hence the tentative referral of OMNH 79032 to *Llistrofus*. The lack of discussion of the feature in *Llistrofus* by past workers may also mean that it was simply overlooked in other taxa. Because many of the grooves seem to lead to pits that are probably nutrient foramina, they may reflect a corresponding soft tissue structure.

The presence of scales is a typical feature of many “microsaurs.” A large number of scales thought to be from the dorsal trunk region are associated with the holotype (Carroll & Gaskill, 1978), and disarticulated scales of a similar form are found in OMNH 79031 (Fig. 12). They are relatively rounded and display radiating striations. Some scales are entirely flat, while others are slightly convex or concave; this may be the product of taphonomic damage or variation along the trunk. This scale morphology is considered to be characteristic of “microsaurs”; similar scales are found in taxa such as *Hyloplesion, Trihecaton*, and *Odonterpeton* (Carroll & Gaskill, 1978) as well as *Microbrachis* (Olori, 2015). Although Carroll & Gaskill (1978:fig. 130D) provided a photograph of the scales of *Llistrofus*, it is not of sufficient quality for accurate comparison with the scales seen in these specimens, and there is no description of the scales. The illustration (Carroll & Gaskill, 1978:fig. 15) more clearly reveals the details and shows that the scales of the holotype similarly consist of a rounded form with a greater width than length and densely packed radiating striations. Many of the scales are broken at the margins when disarticulated. Only a few scales are known from *Hapsidopareion*, while the illustrated scales of *Saxonerpeton* are not of adequate quality for comparisons (Daly, 1973; Carroll & Gaskill, 1978).

Vertebral elements include two complete trunk vertebrae, the atlas and partial axis, an isolated centrum, and four isolated pleurocentra (Movie S1). Given the presence of ribs with the skull and our confidence in associating those elements based on the possible autapomorphic striations of the ribs, it is reasonable to also tentatively associate these vertebrae with *Llistrofus*, but only the atlas-axis complex (Fig. 13; Movie S1) can be confidently associated based on its association with the basioccipital–exoccipital complex. “Microsaurian” trunk vertebrae maintain a fairly generic morphology, with differences pertaining to the contact of the neural arch with the centrum (which likely reflect ontogenetic maturity) and slight deviations in the morphology (e.g., curvature of ventral surface) (Carroll & Gaskill, 1978). In OMNH 79031, a demarcation of a sutural contact is not apparent. The ventral surface is constricted, producing a ridge along the midline. This morphology is in contrast to that of the holotype (Carroll & Gaskill, 1978) and *Hapsidopareion*, in which the sutural contact is still visible (Daly, 1973; Carroll & Gaskill, 1978). This might represent some variation in ontogenetic maturity, but insufficient resolution of the scan to detect a tight suture is also possible. The more incomplete vertebral material is represented by a smaller centrum closely adhered to the vertebra toward the left side of the skull roof.

The atlas is a transversely broad element with a median odontoid that inserts into the concave posterior margin of the basioccipital (Fig. 13). Whether the contact between the neural arch and the centrum is fused or sutured is not clear in OMNH 79031. Another differential feature is whether the two halves of the arches contact each other. In this specimen, they are clearly separated (Figs. 13A and 13B), a feature shared with *Euryodus dalyae* and BPI 3939 (*Micraroter*) (Carroll & Gaskill, 1978). The condition in *Hapsidopareion* is unclear; the element is figured only in lateral profile, and Daly’s (1973) description does not confer any details. Carroll & Gaskill (1978) indicate that they have been separated postmortem. In lateral profile (Fig. 13C), the arches are modestly tall, with a slightly constricted shaft that expands into a subtriangular dorsal head that narrows anteriorly. This differs from that seen in *Hapsidopareion*, in which the dorsal head is shorter in height, bears a slenderer posterior process, and lacks a short, rounded anterior process seen in *Llistrofus* (Daly, 1973; Carroll & Gaskill, 1978). The dorsal head in OMNH 79031 is also expanded transversely, more so medially, such that in dorsal and anterior profiles, it also has a triangular outline, while the shaft appears constricted. The left half is concave dorsally, but the right side is convex; whether this reflects damage to the former, or the close adherence of another element to the latter, is unclear. The axis is mostly lost and cannot be described save for the apparent separation of the halves of the neural arch (Fig. 13). An isolated centrum to the right of the atlas that is smaller than the trunk vertebrae, but of a similar size to the atlas and with a similarly deep excavation of the articular surfaces, may be the detached axis centrum. The pleurocentra are scattered within the block and are of a typical wedge-shaped lateral profile, are concave in dorsal profile, and are generally thin. Confident association with particular vertebrae is not possible.

**Other material.** It must be emphasized that there are a large number of other elements within the block of OMNH 79031, some of which can be confidently excluded from pertaining to any “microsaur” based on size (e.g., phalanges, humerus). Most of the remaining elements are simply flat fragments or are otherwise too poorly preserved to confidently identify either skeletally or taxonomically (Movie S1). Because of the marked disarticulation of some parts of the skull of *Llistrofus* in this block (e.g., right side of the palate, right temporal region), we cannot exclude the possibility that some of the missing elements (e.g., anterior braincase ossifications) are present but are either unidentifiable without a positional context or are not well-preserved; actual loss during preservation also remains possible. A number of extremely thin elements are probably fragmentary scales based on their prevalence on the external surfaces of the block.

Two other elements are noted here because they are of a unique morphology and are paired, which is rare for non-*Llistrofus* elements within this block (Fig. S2). One is positioned ventral to the basal plate of the parasphenoid and the other positioned adjacent to the left quadrate (Fig. 1; Movie S1). These elements are flattened at one end and expand along their length to produce a shallowly indented oval surface that could be considered an articulating facet (Fig. S2). There is also a marked protuberance on the dorsally positioned margin (Fig. S2). The elements cannot represent any part of the cranium, as at least one member of all paired elements is tentatively accounted for. They appear to be broken at the narrower flattened end, and they could represent atlantal ribs, which are typically shorter in length but with more expansion proximally than in other “microsaurs” (Carroll & Gaskill, 1978), or proatlantes, which are not commonly described in recumbirostrans (but see Pardo & Anderson, 2016, for example).

## Discussion

### Morphology of *Llistrofus*

These specimens have contributed several new insights into the anatomy of *Llistrofus pricei*. A number of elements from the skull roof, the palate, the neurocranium, the otic capsule, and the postcranial skeleton are identified and described for the first time in the taxon. This includes information on the previously unknown premaxillae (Fig. S1), which suggest a weakly recumbent snout in *Llistrofus*, and the identification of a septomaxilla in the taxon for the first time (previously its absence was a feature that differentiated it from *Hapsidopareion*). Internal features of the roofing elements (e.g., ventral flange of the prefrontal to buttress the lacrimal, ventral flange of the frontal to contact the orbitosphenoid) were also identified. A short ectopterygoid, similar in relative size to that of *Hapsidopareion* and *Saxonerpeton*, is confidently identified for the first time (Fig. 7). A more complete characterization of the neurocranium (pleurosphenoid, orbitosphenoid, absence of basisphenoid), the otic capsule, and the occiput is also provided.

Additionally, we have confirmed many of the revisions that were made by Bolt & Rieppel (2009). These include: (1) contributions by both the premaxilla and the maxilla to the ventral margin of the external naris, (2) a short overlap of the premaxilla onto the maxilla, (3) 21 maxillary teeth, (4) a contribution of the tabular to the posterolateral margin of the skull table, (5) the absence of palpebral ossifications, and (6) the anterior extension of the surangular to overlap the posterior dentary. We have also noted that the pterygoid, as in the holotype, does not contact the premaxilla due to the transverse extent of the vomers. The new specimens have also contributed additional details and clarity regarding previously described regions of the skull. We have reaffirmed the interesting articulation pattern between paired elements of the medial skull roof in which one will incise into the other across the midline (Fig. 4), which is more developed than in many other “microsaurs.” Other taxa with similar incisions include *Leiocephalikon* (Carroll, 1966:fig. 2)*, Microbrachis* (e.g., Carroll & Gaskill, 1978:fig. 78), and *Euryodus dalyae* (e.g., Carroll & Gaskill, 1978:fig. 68). Most contacts between cranial elements are formed by shallowly angled scarf joints (Fig. 5), a feature that is in contrast to the more widespread interdigitating, abutting, and tongue-and-groove joints seen in recumbirostrans. The clasping of the quadrate between the quadratojugal and the squamosal is also verified in OMNH 79031.

Finally, we documented a few differences between these specimens, the holotype, and previous interpretations of the holotype. Firstly, the two parietals of OMNH 79031 are nearly identical to each other with respect to shape and proportions, in contrast to those of the holotype (Fig. 4), in which they are more asymmetrical (Bolt & Rieppel, 2009). Secondly, the postparietals are more triangular in OMNH 79031, with a more gradual tapering posterolaterally and a straight sutural contact with the parietal, rather than one defined by several interdigitations, as in the holotype (Bolt & Rieppel, 2009). Such intraspecific variation is also evident in the relationship of the median flanges in the midline elements and in the variable presence or absence of enlarged marginal tooth positions. Thirdly, the position and slope of the squamosal is still not fully resolved due to disarticulation in the new specimens (Fig. 2). Its position in OMNH 79031 and in the holotype suggests a more posterior position, following Carroll & Gaskill (1978) but with the posteroventral angle of descent of Bolt & Rieppel (2009). Because the ventral surface of the tabular is smooth, it cannot be determined where the subtemporal flange of the squamosal would sit. Fourth, the cultriform process is not narrowly separated from the basal plate of the parasphenoid in OMNH 79031 (Fig. 7); it appears that this was broken during preservation of the holotype, as the orientation of the separation is slightly oblique (Bolt & Rieppel, 2009), eliminating this character for taxonomic differentiation of hapsidopareiids. Fifth, the angular of OMNH 73718 is extremely narrow ventral to the splenial and only slightly expanded ventral to the prearticular (Fig. 10), whereas the reconstruction of Bolt & Rieppel (2009) shows a taller angular of a more consistent height throughout its length; the element also has a greater lateral exposure than previously reconstructed. Sixth, we have documented a well-developed retroarticular process in both new specimens of *Llistrofus*, particularly in OMNH 79031. Because the mandible of the holotype is partially obscured and disarticulated (Bolt & Rieppel, 2009), it may have been damaged posteriorly in contrast to OMNH 79031 in which the mandibles are encased within the block and completely articulated. Finally, the splenial also appears much shorter anteroposteriorly than reconstructed in the holotype (Bolt & Rieppel, 2009), but as noted in the description, this may be an artifact of segmentation because the element is closely adhered to the angular (Fig. 10).

### Hapsidopareiid taxonomy

*Llistrofus* and *Hapsidopareion* are united mainly on the basis of the large temporal emargination; other features that were originally defined by Carroll & Gaskill (1978) are generic as far as differentiating them from other “microsaurs” (e.g., range of tooth positions in the upper jaw, presence of a pineal foramen) or are impossible to evaluate in one of the taxa (e.g., number of presacral vertebrae in *Hapsidopareion*). A few other features that are shared with a minority of other taxa help to further distinguish the taxon. Examples include the posteroventral expansion of the jugal below the ventral margin of the maxilla (shared with *Pantylus* and *Asaphestera*) and a proportionately small ectopterygoid (shared with *Carrolla* and possibly some gymnarthrids). The new material of *Llistrofus* augments the holotype in providing information about the anterior elements of the possibly recumbent snout, which are absent in the holotype, and reinforces many of the previous findings regarding the morphology of the skull, mandible, and postcrania. We have also identified several features that support the conclusion of Bolt & Rieppel (2009) that *Llistrofus* and *Hapsidopareion* should be retained as separate taxa.

The two taxa were previously differentiated on the basis of the larger size of *Llistrofus*, the contribution of the frontal to the medial orbital margin, and the separation of the cultriform process from the basal plate of the parasphenoid (Bolt & Rieppel, 2009). Size is unreliable unless information is available that indicates that the taxa are of a comparable ontogenetic stage. It is noteworthy that all six specimens of *Hapsidopareion* are of approximately the same size, while all three specimens of *Llistrofus* are of a similar size to each other and are twice as large as *Hapsidopareion*. In the absence of an established ontogenetic series (and poor ontogenetic resolution for most “microsaurs”), it is not possible to resolve in favor of either taxonomy or ontogeny as explanations for the noted differences. As noted above, the separation of the cultriform process from the basal plate is likely the result of taphonomic damage. Although only one original feature (frontal entering the orbit) remains, the new specimens have contributed new data that permit the identification of additional differential features (see the Systematic Paleontology section), such as the separation of the postfrontal from the temporal emargination (Fig. 2) and the absence of a pterygoid–premaxilla contact. Another disparity is the relationship of the cultriform process to the vomers and the premaxillae. In *Llistrofus*, the process contacts the vomers, which separate it from the premaxillae. However, the process in *Hapsidopareion* is excluded from the vomers by the pterygoids (Daly, 1973). The size of the palatal tooth row that extends along the vomer and the palatine and sometimes onto the ectopterygoid relative to the marginal teeth also appears to be informative (though it is unclear whether this is ontogenetic, taxonomic, or a combination). It differs markedly between *Llistrofus* and *Hapsidopareion*, with the latter possessing palatal teeth of a subequal size to the marginal dentition (Daly, 1973; Carroll & Gaskill, 1978), while the former possesses palatal teeth of a notably smaller size and differing distribution (Bolt & Rieppel, 2009). We believe that the taxa should remain separated at present, and that *Hapsidopareion* should be restudied, ideally through CT analysis. However, we reiterate that the disparate size of the two taxa may indicate an ontogenetic explanation for their morphological disparities. For example, the lack of a retroarticular process, the absence of cranial ornamentation, and minor differences in sutural patterns in the smaller *Hapsidopareion* may characterize early ontogeny within the clade. Investigation of the internal anatomy and the degree of ossification of braincase structures would likely contribute informative data to this end.

### Comparisons with other “microsaurs”

The new CT data obtained for *Llistrofus* in this study can be compared with a number of recent works that utilized similar datasets to study other “microsaurian” taxa (Maddin, Olori & Anderson, 2011; Huttenlocker et al., 2013; Pardo, Szostakiwskyj & Anderson, 2015; Szostakiwskyj, Pardo & Anderson, 2015; Pardo & Anderson, 2016). Of note is that all taxa previously sampled with CT are recumbirostrans. As such, these taxa frequently possess features, identified both externally and via tomographic data, that were interpreted to be adaptive for both a fossorial lifestyle and a capability to actively dig or burrow (e.g., consolidation of the posterior braincase, strongly recumbent snout, tall coronoid process). The following comparisons address all regions of the cranium but focus particularly on internal structures such as those of the neurocranium, as much of the new data on *Llistrofus* pertains to this region.

The large temporal emargination results in the most notable differences of the skull roof. The jugal and the postorbital are markedly foreshortened, the tabular has virtually no exposure in lateral profile, and the squamosal is restricted to a tall, slender bar that frames the emargination posteriorly. *Brachydectes* has a similarly large emargination but with a different architecture (i.e., loss of the posterior circumorbital bones) and a more anterior position within the skull, confluent with the orbit (Pardo & Anderson, 2016). Much smaller emarginations are present in many recumbirostrans (e.g., Daly, 1973; Henrici et al., 2011; Huttenlocker et al., 2013; Szostakiwskyj, Pardo & Anderson, 2015). The premaxillae are not fully resolved given the disarticulation or loss in all specimens of *Llistrofus*. Whether the snout was recumbent remains unknown, but it certainly would not have been comparable to the degree seen in recumbirostrans such as ostodolepids. Comparing the sutural contacts is more complicated, as they are sometimes conspicuous in external examination (e.g., extensive interdigitation of *Nannaroter*) and are generally not described in much detail. Most of the sutures in *Llistrofus* are smooth and relatively straight externally, and the majority are simple underplating scarf joints when examined internally (Fig. 5). Complex, interdigitating sutures are often recognized in recumbirostrans (e.g., *Nannaroter, Brachydectes*), and abutting joints are more common in recumbirostrans than in *Llistrofus* (Szostakiwskyj, Pardo & Anderson, 2015). Extensive tongue-and-groove joints were identified in *Carrolla* (Maddin, Olori & Anderson, 2011). The horizontal sections of *Pantylus* (mostly the peripheral snout elements) that were studied by Romer (1969) do not indicate much complexity other than the complex lacrimal morphology that relates to the nasolacrimal duct. The transverse flanges of the median elements in *Llistrofus* are an uncommon feature (but see the frontals of *Nannaroter* and examples cited in the description for other occurrences; Anderson, Scott & Reisz, 2009), but this articulation is still formed by a simple scarf joint. Tighter sutures like those seen in recumbirostrans have often been cited as evidence of adaptation to resist stresses incurred during head-first burrowing (Anderson, Scott & Reisz, 2009; Maddin, Olori & Anderson, 2011).

The palate of *Llistrofus* has been previously well-described on the basis of the holotype (save for the ectopterygoid), and it does not differ significantly from a more generalized “microsaur” beyond relative sizes of the elements. Notably, the ectopterygoid of *Llistrofus*, known only from OMNH 79031 (Fig. 7), is distinctly smaller than the palatine (shared with *Saxonerpeton* and *Hapsidopareion*). Conversely, it is of a subequal size in many other microsaurs. This could be associated with the temporal emargination and the corresponding reduction of adjacent elements. However, *Carrolla* possesses a similarly small ectopterygoid in the absence of any emargination (Maddin, Olori & Anderson, 2011). As noted in our description, gymnarthrids are often reconstructed with an ectopterygoid about a third of the length of the palatine (e.g., Carroll & Gaskill, 1978:fig. 109), but this region is rarely exposed in most gymnarthrid specimens.

The most relevant features regarding the mandible pertain to the development of the coronoid process and the retroarticular process. The coronoid process in most recumbirostrans is much taller than in *Llistrofus*, in which it barely rises above the level of the tooth row (Fig. 10). Many other basal “microsaurs” (e.g., *Asaphestera*; Carroll, 1963) also possess comparably low processes. Some of the more enigmatic (often Carboniferous) taxa that would be considered recumbirostrans by their historical classification but that have not been incorporated into a phylogenetic matrix to test their position (e.g., the gymnarthrids *Sparodus* and *Leiocephalikon*) also possess short processes (Carroll, 1966). A developed process has been cited as adaptive for feeding within burrows by increasing the attachment surface to accommodate enlarged adductor musculature in recumbirostrans (Szostakiwskyj, Pardo & Anderson, 2015). This is also seen in some extant fossorial tetrapods (Rieppel, 1981; Pregill, 1984). It should be noted that nonfossorial tetrapods may also have a tall process as a result of other selective pressures such as in many extant mammals (Pérez-Barbería & Gordon, 1999), and more work is required to evaluate this hypothesis. In a similar vein, expansion of the *M. depressor mandibulae* and its insertion onto a correspondingly enlarged retroarticular process has been cited as adaptive for mitigating the constraints of feeding in confined spaces (Pardo & Anderson, 2016). Both of the new specimens of *Llistrofus* do possess a retroarticular process, being more developed than in many non-recumbirostran “microsaurs,” such as *Asaphestera* (Carroll, 1966) and *Hapsidopareion* (Daly, 1973). In the holotype of *Llistrofus*, the mandible terminates abruptly in a squared-off end (similar to *Dvellecanus*; Szostakiwskyj, Pardo & Anderson, 2015). Because the new specimens of *Llistrofus* are comparably sized to the holotype, disparity in ontogenetic maturity seems to be an unlikely explanation, although it cannot be ruled out. Taphonomic damage to the holotype, in which the mandible is partially disarticulated and partially obscured, may also play a role. The process is relatively stout in OMNH 79031 and is less elongate than in *Aletrimyti* and *Brachydectes* (Szostakiwskyj, Pardo & Anderson, 2015; Pardo & Anderson, 2016). Not all recumbirostrans possess a retroarticular process; it is absent in *Huskerpeton* (Huttenlocker et al., 2013), *Dvellecanus* (Szostakiwskyj, Pardo & Anderson, 2015), and *Carrolla* (Maddin, Olori & Anderson, 2011), for example. Other minor differences include the far-reaching extent of the angular at the expense of the splenial in *Llistrofus* (which we reiterate is not fully or confidently resolved in any specimen) and the presence of a Meckelian foramen.

The neurocranium of *Llistrofus* is characterized by a lesser degree of ossification relative to recumbirostrans (Fig. 9). Although many of the same ossifications are found in both *Llistrofus* and recumbirostrans (e.g., orbitosphenoid), the degree of ossification seen in *Llistrofus* is relatively low. For example, the orbitosphenoid of *Llistrofus* forms a square in lateral profile (Figs. 9G–9J), and it is widely separated from the antotic region. In many of the more generalized recumbirostrans (e.g., *Huskerpeton, Dvellecanus, Nannaroter, Pantylus*), the orbitosphenoid is anteroposteriorly elongate with a rectangular lateral profile (Romer, 1969; Huttenlocker et al., 2013; Szostakiwskyj, Pardo & Anderson, 2015). In some of these taxa (e.g., *Dvellecanus, Rhynchonkos*), it closely approaches the pleurosphenoid posteriorly to frame the foramen for the oculomotor nerve. On a related point, the rectangular orbitosphenoid of other taxa may maintain its height such that it contacts ventral flanges of both the frontal and the parietal (e.g., *Dvellecanus, Aletrimyti*), sometimes jointly framing a circular depression on the underside of the roofing elements (e.g., *Rhynchonkos*) (Szostakiwskyj, Pardo & Anderson, 2015). In other instances, the orbitosphenoid underlies the parietal but does not contact it (e.g., *Nannaroter, Micraroter*) (Szostakiwskyj, Pardo & Anderson, 2015). Both are in contrast to *Llistrofus* in which the orbitosphenoid neither underlies the parietal nor contacts it; no ventral flange is present on the parietal. Increased contact between the skull roof and braincase would presumably act as a bracing mechanism, but the significance of the variance seen in recumbirostrans remains to be explored. The separation between the orbitosphenoid and the antotic region is also seen in brachystelechids (to a lesser degree in *Quasicaecilia*) (Maddin, Olori & Anderson, 2011; Pardo, Szostakiwskyj & Anderson, 2015) and in *Brachydectes* (Pardo & Anderson, 2016). Interestingly, several anterior braincase ossifications that are seen in recumbirostrans are not identified in any specimen of *Llistrofus*, but there is also great variance among recumbirostrans with respect to the presence and nature of these ossifications. These include median elements identified as the presphenoid, a posterior ossification of the ethmoid trabeculae, and the mesethmoid (Pardo, Szostakiwskyj & Anderson, 2015). Whether this absence is related to phylogeny or to ontogeny is unclear.

A final noteworthy difference is the absence of a large dorsal sinus between the supraoccipital and the postparietals/parietals in *Llistrofus*. Sinuses of variable size are described in some recumbirostrans, such as *Dvellecanus* and *Aletrimyti* (Szostakiwskyj, Pardo & Anderson, 2015), *Huskerpeton* (Huttenlocker et al., 2013) and the ostodolepids *Pelodosotis* (Carroll & Gaskill, 1978), and *Nannaroter* (Anderson, Scott & Reisz, 2009). This is accomplished by some combination of vaulting of the posterior skull table (particularly in ostodolepids) and a posterior shift in the supraoccipital (compared to other Paleozoic tetrapods) such that it has a distinct dorsal exposure. The supraoccipital of both the type specimen and OMNH 79031 (Fig. 1) is well exposed dorsally, but there is no vaulting of the posterior skull table (Figs. 1 and 2). As a result, virtually no gap exists between the anterior extent of the supraoccipital and the roofing elements, similar to the condition described in *Rhynchonkos* and *Brachydectes* (Szostakiwskyj, Pardo & Anderson, 2015; Pardo & Anderson, 2016).

### Phylogenetic analysis

Based on the wealth of new data obtained through the tomographic analysis, we re-examined *Llistrofus* using phylogenetic matrices of previous workers (Huttenlocker et al., 2013; Pardo et al., 2017). Our reanalysis recovered identical tree topologies to those found in those previous studies. Because the topologies and phylogenetic position of *Llistrofus* remained unchanged, we do not present the full details or a visualization of the results here. The revised character codings for *Llistrofus* are available for future workers in the Supplemental Information. Below is a brief summary of the most relevant information.

In the original analysis of Huttenlocker et al. (2013), only *Hapsidopareion* and *Saxonerpeton* were included, and they were recovered as sister taxa. Our inclusion of *Llistrofus* produced the intuitive recovery of *Llistrofus* and *Hapsidopareion* as sister taxa (supported by 90% bootstrap support) with *Saxonerpeton* as the sister taxon to this grouping. The node joining *Hapsidopareion* and *Saxonerpeton* was supported by 59% bootstrap support in the original analysis, and the addition of data for *Llistrofus* slightly increased for the grouping of Hapsidopareiidae + *Saxonerpeton* (60%). This clade remains as the earlier diverging sister group to recumbirostrans. This result is mirrored in the reanalysis of the Pardo et al. (2017) matrix in which *Llistrofus* is the only one of the above three taxa that is included. Its previously recovered position as diverging earlier than recumbirostrans (Pardo et al., 2017:extended data fig. 7) is again recovered here. Support for monophyly of the sampled “microsaurs” remains strong (83% bootstrap support). Considering the hapsidopareiids’ phylogenetic position, the morphology and associated ecology of *Llistrofus* may represent an earlier stage in the step-wise acquisition and development of recumbirostran characters that are seen in more conspicuously modified taxa. For example, increased ossification of the braincase (primarily the orbitosphenoids in *Llistrofus*) and increased dorsal exposure of the supraoccipital may be trends in “recumbirostran” evolution that are captured here. One of the main limitations in a better understanding of the phylogenetic framework is that most of the earliest “microsaurs” (e.g., *Asaphestera, Tuditanus, Crinodon*) preserve very little of the neurocranium. Most specimens are flattened and obscure both three-dimensional data and inner structures (Carroll, 1966; Carroll & Gaskill, 1978). None have been analyzed via tomographic methods, and as a result, contextualizing the neurocranial architecture (amongst other details) of *Llistrofus* can only be made against the “upper-bound” of the recumbirostrans. It is important to note that the limited number of specimens for most “microsaurian” taxa and a lack of comparable sampling of the earliest “microsaurs” restricts our ability to differentiate between their possible phylogenetic positions (relatively basal) and their ontogenetic immaturity. These hypotheses cannot be easily or readily teased apart at present, underscoring the fundamental challenges associated with interpretations of unusual morphologies in extinct taxa. Taphonomic loss of some elements, although unlikely for OMNH 79031, also cannot be excluded.

### Interpretations of the temporal emargination

As previously noted, the emargination of *Llistrofus* is equaled in size only by that of *Brachydectes* in which the absence of a postorbital bar leads it to be confluent with the orbit. Previous workers (Szostakiwskyj, Pardo & Anderson, 2015; Pardo & Anderson, 2016) suggested that the emargination in various recumbirostrans would accommodate expansions of the adductor musculature to mitigate the reduced mechanical advantage that is associated with feeding within constrained spaces. Other hypotheses were previously suggested for *Llistrofus* by Bolt & Rieppel (2009), including cranial kinesis, a durophagous ecology, and the absence of any functionality. Here, we discuss these hypotheses in the context of the unusual emargination seen in *Llistrofus*.

The concept of cranial kinesis is perhaps most readily appealing because a reduction of bones in the temporal region is often correlated with kinetic skulls in extant squamates (Herrel et al., 2007). The observation that most of the cranial sutures are scarf joints further suggests a relatively loose skull. However, fenestration in squamates is often taken to a greater extreme than what is seen in *Llistrofus* (e.g., substantial reduction or loss of the postorbital bar), such as in many geckos and in varanids (Frazzetta, 1962; Herrel et al., 1999). Beyond the cursory mention of kinesis in *Llistrofus* by Bolt & Rieppel (2009), kinesis is rarely considered in the “microsaurian” literature. These authors proposed that it was unlikely due to the apparent absence of mobile joints in the skull, underscoring the point that relatively loose sutures like the observed scarf joints do not necessarily imply mobile sutures. Bolt & Wassersug (1975) discussed the potential for kinesis in *Lysorophus* but suggested that modifications seen in the skull were likely the product of locomotory pressures such as burrowing and swimming, rather than kinesis. The inherent limitations in inferring such attributes from hard tissue alone further complicate the matter. As noted by previous workers (Holliday & Witmer, 2008), both the presence of inferred morphological correlates of kinesis and phylogenetic bracketing in extant animals may be misleading when measured against observational data of those taxa. Because there is no established precedent (e.g., biomechanical studies) with respect to “microsaurs” that would strongly support a hypothesized kinetic skull, it seems best to leave the question as unresolved.

There is more precedent (Szostakiwskyj, Pardo & Anderson, 2015; Pardo & Anderson, 2016) for suggesting that the emargination could have accommodated expanded adductor musculature (specifically *mm. adductores mandibulae*), an adaptation to mitigate the reduced mechanical advantage associated with feeding in confined spaces. Whether the musculature was always expanded or whether it was simply accommodated into a different spatial configuration remains unclear. Hapsidopareiids in general lack a tall coronoid process, often seen in recumbirostrans, that has been interpreted to allow for insertion of enlarged muscles, again to mitigate constraints of confined spaces, in the same vein as in amphisbaenians (Szostakiwskyj, Pardo & Anderson, 2015). This would suggest by relative comparison that *Llistrofus* did not have greatly enlarged musculature. As noted by Bolt & Rieppel (2009), the low process also provides counterevidence to any hypothesized duropaghy; this is supported by a lack of dental modifications often associated with this ecology in other “microsaurs” (e.g., *Pantylus*; Romer, 1969). The accommodation of musculature was previously suggested in lysorophians (Bolt & Wassersug, 1975; Wellstead, 1991) and more widely in emarginated lepospondyls under an inferred close relationship to lissamphibians (Carroll & Holmes, 1980). A possible modern analogue may be found in caecilians, which have been shown in one experimental study to not exhibit significant difference in burrowing performance between fenestrated and nonfenestrated skulls (Kleinteich et al., 2012); the authors suggested that fenestration could be more related to the arrangement of muscles than to burrowing performance. Again, we reiterate that the poor understanding of muscle arrangement in extinct taxa makes it difficult to more fully evaluate this hypothesis.

Possibly concurrent with accommodation of musculature is the reorganization of both soft and hard tissues in the cranium that is frequently associated with miniaturization (Hanken & Wake, 1993). Miniaturization has been more thoroughly explored in the coeval amphibamid temnospondyls, with a focus on how this process could relate to the origin of modern lissamphibians (Fröbisch & Schoch, 2009; Schoch, 2013; Pérez-Ben, Schoch & Báez, 2018). With respect to “microsaurs,” miniaturization has been discussed with respect to the smaller brachystelechids *Carrolla* (Maddin, Olori & Anderson, 2011) and *Brachydectes* (Pardo & Anderson, 2016). Based on those taxa, there is not much evidence at present to suggest that the emargination of *Llistrofus* is associated with miniaturization. Firstly, *Llistrofus* is larger than brachystelechids and is of a more comparable size to many other “microsaurs” (e.g., gymnarthrids), including nonemarginated taxa (e.g., *Asaphestera*). It thus seems somewhat implausible that such a massive emargination would be associated with a diminutive body size when many comparably sized “microsaurs” exhibit no such adaptation. Secondly, miniaturization is typically accompanied by consolidation and hyperossification (Hanken & Wake, 1993). This is found in the brachystelechids, with their co-ossified posterior brains (Maddin, Olori & Anderson, 2011; Pardo, Szostakiwskyj & Anderson, 2015), but these taxa are also some of the smallest “microsaurs.” *Llistrofus*, however, lacks any degree of consolidation or co-ossification.

Paedomorphism or other heterochronic events may also be considered as an explanation for the emargination (i.e., reduction of elements) and the neurocranium (i.e., absence of comparable ossification to recumbirostrans). Although unlikely, it is not possible to exclude this hypothesis given the present paucity of ontogenetic studies that would allow for an identification of juvenile features. The ornamentation is comparable to that of juveniles of *Microbrachis* (Olori, 2015), but this taxon is aquatic and may have had different constraints imposed on its development compared to terrestrial “microsaurs.” Most emarginated “microsaurs” (e.g., *Nannaroter*, *Tambaroter*) are known from only the holotype or from a handful of specimens of a similar size; *Llistrofus* and *Hapsidopareion* are the same in this regard. The only evidence for any change to the emargination in any “microsaur” is the loss of a small emargination in juveniles of *Cardiocephalus peabodyi* during maturation (Anderson & Reisz, 2011). There is no evidence that the *Llistrofus* specimens belonged to relatively mature individuals that possessed a large emargination at a juvenile stage. Especially if *Llistrofus* is a more mature form of *Hapsidopareion*, then there is no evidence for a change in the relative size or construction of the emargination within this limited ontogenetic range. Furthermore, the epipodials are ossified in the holotype of *Llistrofus*, and the septomaxilla is well-ossified in OMNH 79031, which would be unexpected in a paedomorphic form. Finally, all known specimens of *Llistrofus* are within the same size range as many generalized recumbirostrans.

In summary, the functional and evolutionary driver(s) of the hapsidopareiid temporal emargination remain unresolved. Several factors may be at play, including ones that have not been addressed here. Interpretations of modifications to cranial architecture based strictly on hard tissues is a challenging and sometimes misleading exercise in extant taxa, and the absence of soft tissue and observational data to further evaluate hypotheses for extinct taxa only further compounds the matter. A possible future direction that could shed some additional light on soft tissue (i.e., muscle attachments) would be serial thin sectioning of cranial elements, particularly around the emargination (and perhaps of the mandible) to examine osteological correlates for attachment (Hieronymus, 2006; Payne, Holliday & Vickaryous, 2011; Petermann & Sander, 2013).

### Proposed ecology of *Llistrofus*

Inferring the paleoecology of extinct organisms is often a challenging task, particularly with respect to more unusual morphologies or cryptic lifestyles. Nonetheless, some proposals may be put forth regarding the paleoecology of *Llistrofus* in light of our current understanding of “microsaurs” and Paleozoic tetrapods at large. In general, *Llistrofus* possesses an interesting mixture of features, including some attributes that were previously associated with fossoriality in various recumbirostrans. For example, the orbitosphenoids form a firm abutting joint with the ventral flange of the frontal, and the antorbital rim is reinforced by a ventral process of the prefrontal that buttresses the dorsal process of the lacrimal. This form of bracing of the roof elements through contact with either palatal or neurocranial elements (or both) has been suggested to help resist directional stresses in recumbirostrans in which such contacts are more common and more developed (Szostakiwskyj, Pardo & Anderson, 2015). These articulations may have helped to resist directional stresses. The supraoccipital is exposed dorsally, possibly for additional insertion of epaxial musculature (Szostakiwskyj, Pardo & Anderson, 2015). The holotype of *Llistrofus* preserves a large portion of the postcrania, including reduced forelimbs and a relatively elongate torso. These are attributes that are identified in other “microsaurs” that are inferred to have been fossorial (Szostakiwskyj, Pardo & Anderson, 2015) as well as in extant taxa (Gans, 1974), although these features may also appear through other selection pressures and in other environments. However, many of the cranial sutures in *Llistrofus* are simple, relatively loose scarf joints, in contrast to interdigitating, abutting, and tongue-and-groove joints commonly observed in recumbirostrans that are presumed to have been for resisting various stresses associated with head-first burrowing (Maddin, Olori & Anderson, 2011). The coronoid process of *Llistrofus* is low, more comparable to the plesiomorphic condition than to that of any recumbirostran in which a tall process is considered to be adaptive for feeding in confined spaces. Elements of the occiput and the posterior braincase of *Llistrofus* are not partially (e.g., the occiput of *Dvellecanus*) or fully co-ossified (as in brachystelechids) (Maddin, Olori & Anderson, 2011; Pardo, Szostakiwskyj & Anderson, 2015; Szostakiwskyj, Pardo & Anderson, 2015). Collectively, this presents a confusing jumble of features in *Llistrofus*, some of which are correlated with fossoriality and/or active burrowing and some of which are contrary to these correlations (especially to active burrowing). Here, we propose that *Llistrofus* was a fossorial taxon in the sense of living underground or within substrate, but that it was relatively incapable of more active burrowing compared to taxa with consolidated skulls. *Llistrofus* may have occupied “subterranean” settings that would be readily accessible without requiring active tunnel construction or expansion (e.g., leaf litter, existing abiogenic, or biogenic tunnels). This is perhaps one of the most difficult cryptic ecologies to demonstrably support in the fossil record; even most recumbirostrans are not preserved in burrows, but it may be reasonably inferred and articulated based on a brief analogue with extant fossorial taxa.

The nuanced ecology we propose is widely observed among extant fossorial taxa, although it can encompass a broad range of specific ecologies. On one end of the range (opposite to that of active burrowers) are organisms that simply live beneath various objects or coverings on the surface (e.g., leaf-litter, fallen trees, rocks). Among vertebrates, reptiles and amphibians are particularly common occupants of leaf-litter settings (Fauth, Crother & Slowinski, 1989; Vonesh, 2001). A step up from this is are taxa that may utilize more physically defined structures that were formed through various processes. These may be either abiogenic, such as cracks formed by aquatic or aeolian processes, or biogenic, such as the appropriation of structures made by other organisms (e.g., see Kinlaw & Grasmueck, 2012 for a specific example; see Jones, Lawton & Shachak, 1994 for a broader survey). Finally, some organisms may be capable of propagating existing structures (passive burrowing) but may be relatively inefficient at or incapable of creating their own burrows (e.g., the salamander *Ambystoma*; Semlitsch, 1983). It is not possible to determine which of these fossorial, nonactive burrowing ecologies *Llistrofus* might have occupied, but these examples are presented to illustrate the diversity of fossorial ecologies that could be plausibly applied to our interpretation.

The proposed fossoriality of *Llistrofus* also contextualizes the taxon within the diverse early Permian assemblage preserved at the Richards Spur locality. Small-bodied taxa are typically rare in the early Permian, largely because many of the preserved environments are higher energy fluvial or floodplain settings that impose a size-related preservation bias. The particular nuances of deposition at the locality (aqueous mobilization of surficial remains into the fissures and the natural entrapment of living animals; MacDougall et al., 2017) suggest that hydrodynamic sorting is not a major filter in this instance. Other small-bodied tetrapods such as the amphibamid *Doleserpeton* are among the most abundant taxa (MacDougall & Reisz, 2017). The gymnarthrid *Cardiocephalus peabodyi* is not as common as those taxa, but it is more abundant than either *Llistrofus* or *Nannaroter* (Carroll & Gaskill, 1978). Anderson, Scott & Reisz (2009) proposed that the paucity of *Nannaroter* (known only from the holotype at the time) reflected either a natural paucity in the environment or the generally low preservation potential associated with fossorial ecologies. *Llistrofus* is now known only from three skulls and remains a similarly rare taxon. The proposed fossorial ecology is certainly one plausible explanation for their paucity. In some instances, fossorial ecologies are documented to facilitate preservation of taxa in more recent karst deposits (Cassiliano, 1997) because they are essentially buried immediately and are not exposed to adverse surface conditions such as scavengers and weathering. However, karst deposits formed by mobilization or entrapment typically suffer from a paucity of more specialized taxa (scansorial, arboreal, fossorial) (Reed, 2006), conditions that are similar to the Richards Spur locality (MacDougall et al., 2017). The vertebrate record of the locality thus may reflect an ecological filter, at least in part, rather than a size or a hydrodynamic filter that is often responsible for taphonomic biases in the more widespread early Permian floodplain deposits. A natural paucity in the environment that is independent of ecology to a degree cannot be excluded.

## Conclusion

Here, we have presented new data regarding the hapsidopareiid *Llistrofus pricei* that have greatly improved our knowledge of its unusual anatomy. This allows for a fuller discussion of the large temporal emargination in hapsidopareiids, the relationship between *Llistrofus* and *Hapsidopareion*, and some inferences of the potential fossorial paleoecology of this taxon. At present, *Llistrofus* and *Hapsidopareion* should be maintained as distinct taxa, but this is partly because of relatively poor preservation of the latter and great uncertainties regarding “microsaur” ontogeny in general. We have postulated that *Llistrofus* was fossorial but not capable of active burrowing. Differences from the recumbirostran morphology, such as the absence of ossifications of the anterior braincase and the posteriorly truncated orbitosphenoid, and the temporal emargination cannot be confidently explained by any particular factor (e.g., phylogeny, ontogeny, ecology) and could represent a multiplicity of factors involved with the evolution of this intriguing lineage of diminutive, fossorial taxa. Continued work on “microsaurs” and other Paleozoic tetrapods will be necessary to further test hypotheses of paleoecology and to better understand the range of morphological and ontogenetic variation within the clade. The paucity of *Llistrofus* at Richards Spur, as with *Nannaroter*, can be reasonably inferred to be, in part, the product of preservational attributes of the locality (MacDougall et al., 2017).

This study also demonstrates the efficacy of NT of vertebrate fossils from the karst deposits of Richards Spur. The total number of elements that could be identified through the CT data greatly exceeds that which could be identified from surficial exposures alone, and many of these otherwise hidden elements have contributed important new information regarding the neurocranium and additional nuances and details of previously lesser known regions of the skull roof and palate. Material from Richards Spur is commonly concentrated in dense, multitaxic blocks, and preparation is often complicated by disarticulation and mixing of skeletal elements and by the fragility of small material. These factors have often limited detailed studies, especially when specimens of focal interest are preserved with numerous other elements, as in OMNH 79031. Metallic minerals, particularly pyrite, that commonly interfere with processing of X-ray CT data, are also abundant. These challenges are mitigated by NT, as neutron beams experience minimal attenuation when passing through metals, and the method is further enhanced with material from the locality due to the enrichment of fossils with hydrocarbons, as neutron beams interact highly with light organic compounds (Schwarz et al., 2005; Sutton, 2008; Mays et al., 2017). The ability to utilize this method to evaluate and to reevaluate specimens from the locality may thus produce a large body of new data of the taxa preserved at the site.

## Supplemental Information

10.7717/peerj.6327/supp-1 Supplemental Information 1 Figure S1. Selected profiles of the left quadrate of OMNH 73931. (A) Anterior profile; (B) lateral profile; (C) posterior profile; (D) medial profile; (E) ventral profile; (F) dorsal profile. Scale bars equal to 1 mm.

10.7717/peerj.6327/supp-2 Supplemental Information 2 Figure S2. Selected profiles (tentatively identified) of possible atlantal rib or proatlas. (A) Posterior profile; (B) lateral profile; (C) anterior profile; (D) proximal profile. Scale bar equal to 1 mm.

10.7717/peerj.6327/supp-3 Supplemental Information 3 Movie S1. Animation representing the step-wise digital segmentation of OMNH 79031. The animation progresses through six complete rotations, with an increased filtering with each cycle: (1) volume rendering of the block containing OMNH 79031; (2) isosurface generated at a threshold value of 145; (3) rendering of all material associated with *Llistrofus*; (4) rendering of the entire cranium and mandibles of OMNH 79031; (5) rendering of the previous step without the mandibles; (6) rendering of the palate, neurocranium, and otic capsule; (7) rendering of the previous step without the palate. Color palettes correspond to those of the in-text figures.

## Institutional Abbreviations

FM: Field Museum, Chicago, IL
OMNH: Sam Noble Museum of Natural History, Norman, OK.

## Additional Information and Declarations

### Data Availability

The following information was supplied regarding data availability: Unprocessed 16-bit TIFF slices are available online through MorphoBank (project #3134, http://morphobank.org/permalink/?P3134).
