# Supplementary figures and images for: New material of the ‘microsaur’ Llistrofus from the cave deposits of Richards Spur, Oklahoma and the paleoecology of the Hapsidopareiidae
Source: PeerJ. 2019 Jan 25;7:e6327. doi: 10.7717/peerj.6327 (PMC6348957; doi:10.7717/peerj.6327)

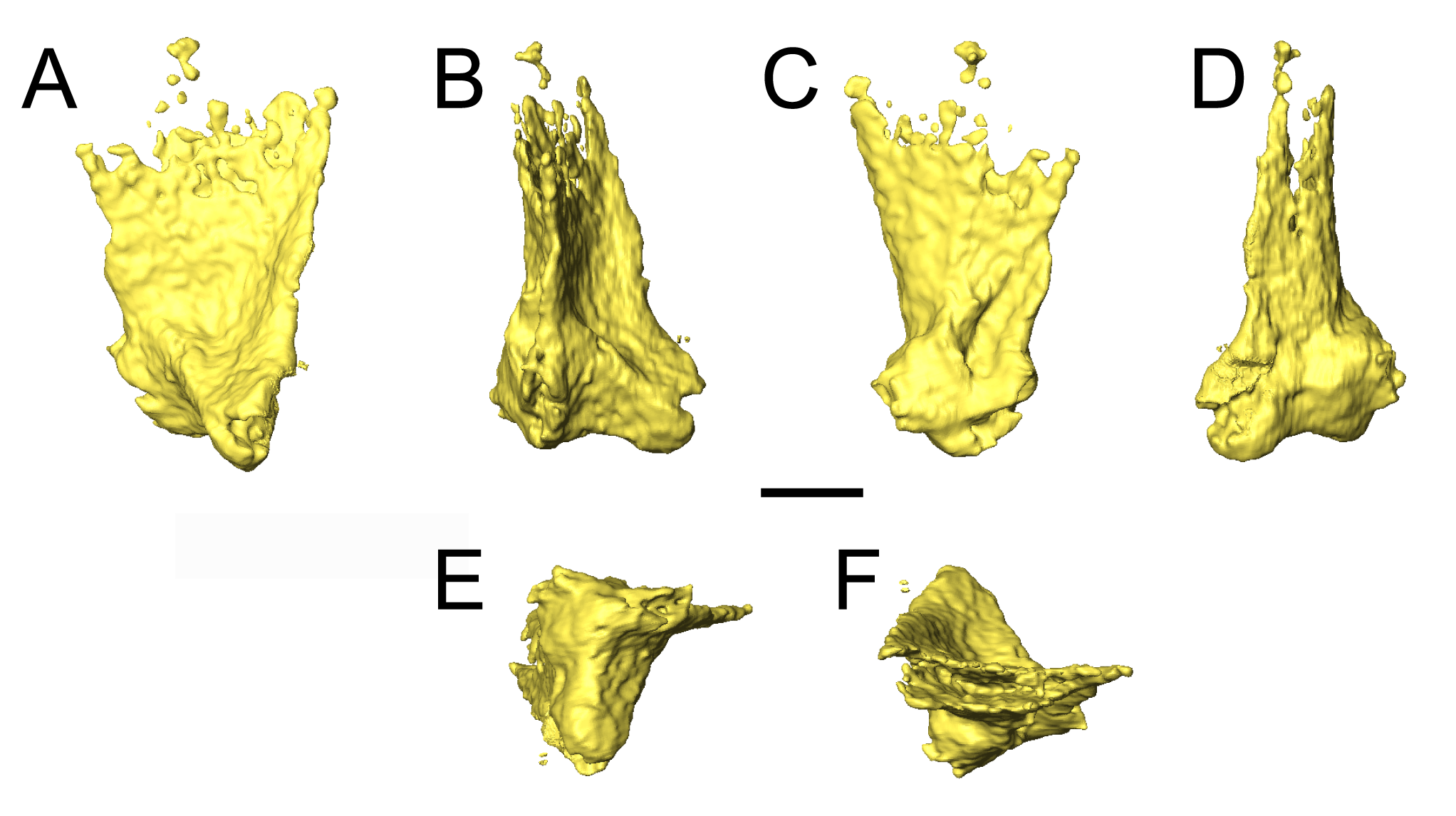

Supplement: Supplemental Information 1 — (A) Anterior profile; (B) lateral profile; (C) posterior profile; (D) medial profile; (E) ventral profile; (F) dorsal profile. Scale bars equal to 1 mm. [file peerj-07-6327-s001.png]

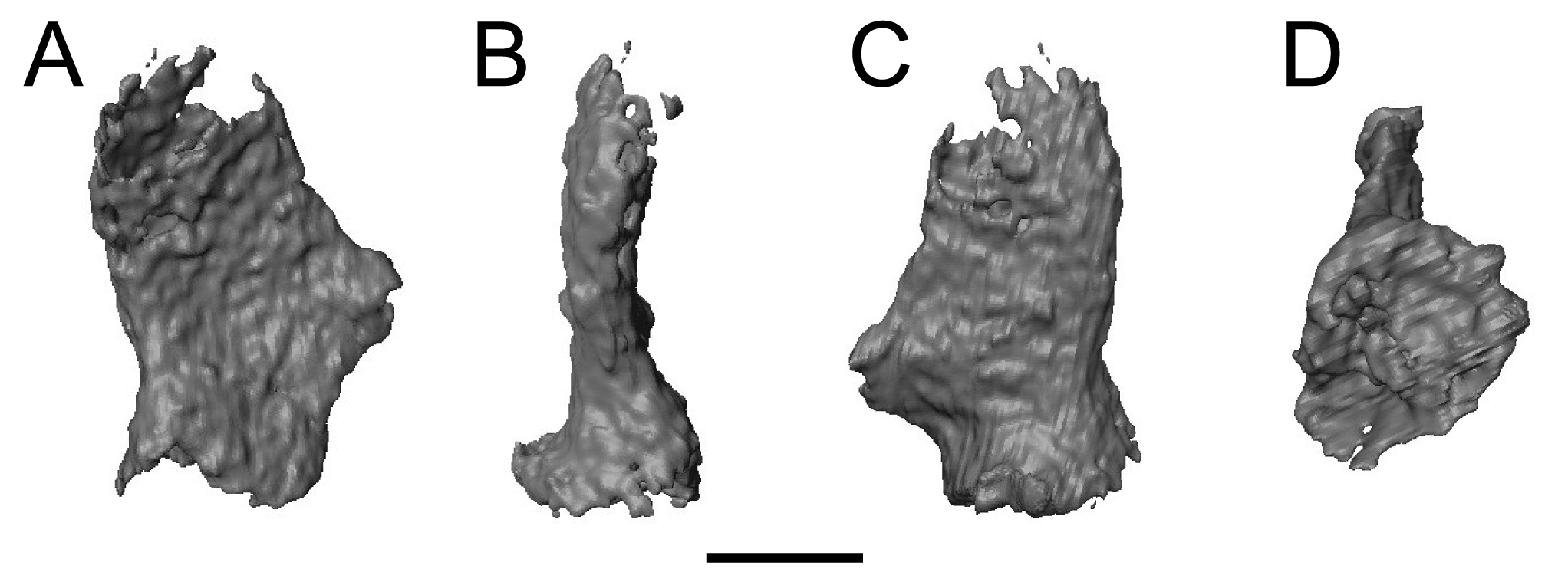

Supplement: Supplemental Information 2 — (A) Posterior profile; (B) lateral profile; (C) anterior profile; (D) proximal profile. Scale bar equal to 1 mm. [file peerj-07-6327-s002.png]
